# Early colonization before inundation consistent with northern glacial refugia in Southern Doggerland revealed by sedimentary ancient DNA

**DOI:** 10.1073/pnas.2508402123

**Published:** 2026-03-10

**Authors:** Robin G. Allaby, Rosie Ware, Rebecca Cribdon, Teri A. Hansford, Tim Kinnaird, Derek Hamilton, Logan Kistler, Phil Murgatroyd, Richard Bates, Simon Fitch, Vincent Gaffney

**Affiliations:** ^a^Faculty of Science, Engineering and Medicine, School of Life Sciences, Gibbet Hill Campus, University of Warwick, Coventry CV4 7AL, United Kingdom; ^b^Faculty of Science, School of Earth and Environmental Sciences, University of St. Andrews, St. Andrews KY16 9AL, United Kingdom; ^c^Faculty of Management, Sciences and Engineering, Scottish Universities Environmental Research Centre Radiocarbon Dating Laboratory, Scottish Enterprise Park, East Kilbride G75 0QF, United Kingdom; ^d^Department of Anthropology, National Museum of Natural History, Smithsonian Institution, Washington, DC 20560; ^e^School of Archaeological and Forensic Sciences, University of Bradford, Bradford BD7 1DP, United Kingdom

**Keywords:** sedaDNA, Mesolithic, Pterocarya, inundation, refugia

## Abstract

Prior to the formation of the present-day North Sea during the mid-Holocene, North-Western Europe was connected through the Doggerland landmass. While it has been known for the past century that Doggerland was forested, it has not been clear when the onset of forestation occurred or whether the environment was more habitable for humans than surrounding European areas. In this study, we reconstruct the paleoecology of a river system, the Southern River, from the late Late Pleistocene to the late Holocene using sedimentary ancient DNA (sedaDNA) from 252 sediment samples from 41 cores spanning the length of the river system and headwater area. We identify secure and insecure sedaDNA signals by integrating sedimentological and sedaDNA data into a taphonomic model. Secure sedaDNA signals are found in silty and fine sand deposits where 95 to 98% originates from local deposition, but coarse sands and gravels are insecure with 60 to 70% of the sedaDNA associated with mixed ecosystem signals from reworked and influxed sediments. Secure sediments reveal the presence of several temperate tree genera such as *Quercus*, *Ulmus,* and *Corylus* over 16,000 y ago in the Late Pleniglacial, and thermal indicator genus *Tilia* several thousand years earlier than has been recorded for surrounding European areas. In this area, we also detect an anomalous signal of the genus *Pterocarya*, considered extinct in the region since the Hoxnian Stage (~400 ka). These observations are consistent with colonization from nearby northern glacial refugia, suggesting a favorable environment in which the cultural Mesolithic could develop.

## Significance

The Doggerland landmass connected North-Western Europe during the Late Pleistocene (approximately 129 to 11.7 ka) and Early Holocene (approximately 11.7 to 8.2 ka) and was likely a key area for Mesolithic peoples. In this study, we show the early presence of temperate species including a species thought extinct, indicating a likely close proximity of refugia with important resource implications for Mesolithic peoples. We also show that ecological turnover combined with sediment turnover can be used to understand the taphonomic processes leading to sedimentary ancient DNA (sedaDNA) deposition and reworking. Using this approach, we show that in alluvial systems fine sediments are associated with secure deposits, but more sandy deposits are at greater risk of giving mixed ecological profiles through reworked sedaDNA.

## Doggerland as a Mesolithic Landscape

**Fig. 1.**
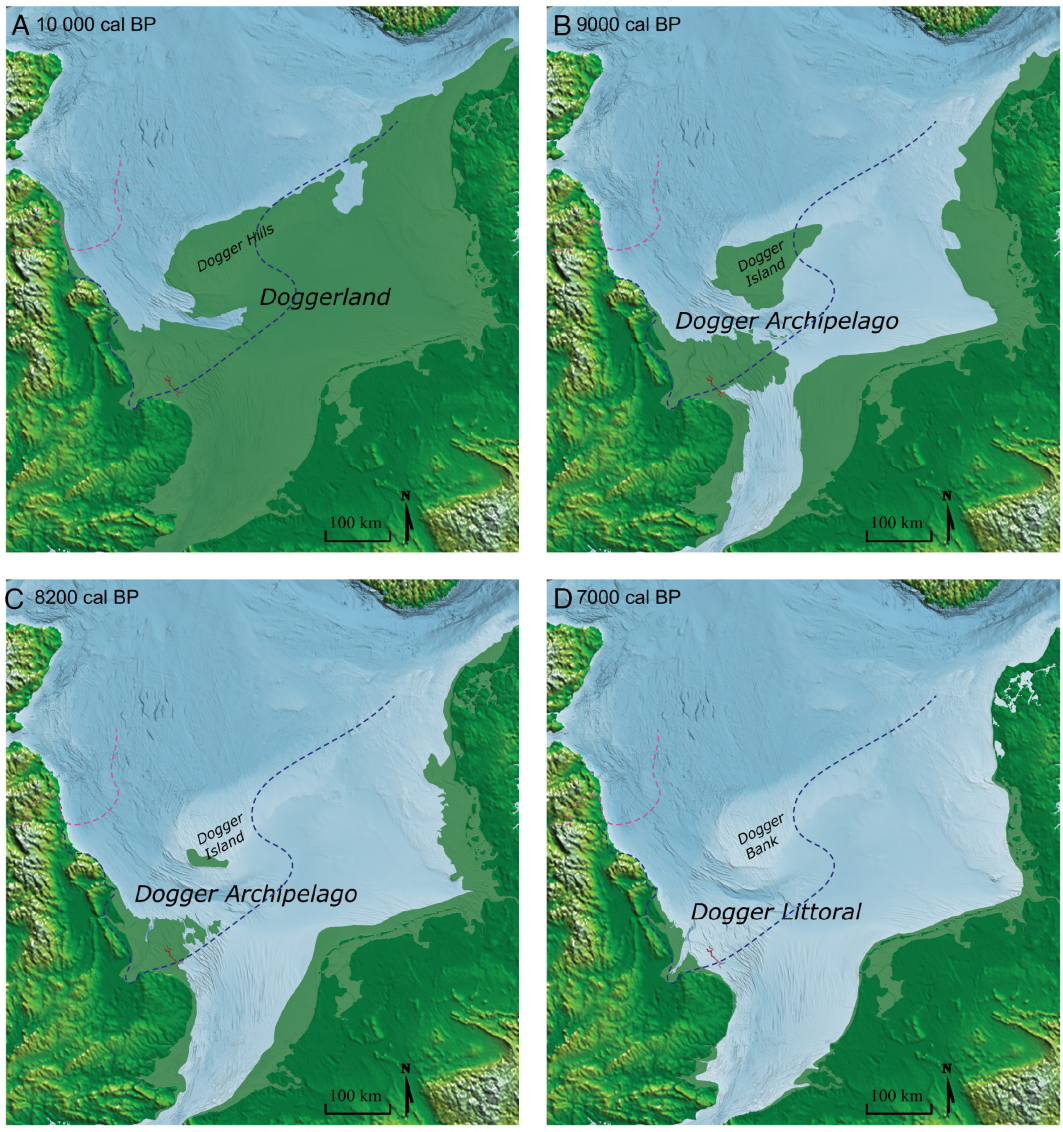
Holocene Doggerland coastline reconstructions in relation to the Southern River. Coastline reconstruction at A. 10000 cal BP, B. 9000 cal BP, C. 8200 cal BP and D. 7000 cal BP. Southern River shown in red. Maximum ice advances shown for 18.4-17.3 ka (dotted line in pink) and 25.8-24.6 ka (dotted line in blue) (14). Figure adapted from Walker et al. 2020 (10).

During the Late Pleistocene (approximately 129 to 11.7 ka) and early Holocene (approximately 11.7 to 8.2 ka), prior to the formation of the present-day North Sea, North-Western Europe was connected through a low undulating landmass known as Doggerland (1). The landscape was for a time forested (2, 3), and likely to have been important for Mesolithic communities by providing a resource-rich environment as evidenced by bone and antler artifacts indicating the presence of hunting cultures (4–7). The most celebrated of these artifacts is the Colinda harpoon (4), attributable to the Maglemosian culture that existed in North-Western Europe from around 10.3 ka (8). This environment persisted until the mid-Holocene after which episodic sea level rise inundated the landscape (9, 10). It is still the case that very little is known of the role this environment may have played during the last glaciation in terms of providing habitable locations or when it could have been first colonized by forests and people. During the Devensian glacial advances, a complex confluence of British and Fennoscandian ice sheets met on Doggerland (11, 12), which drained into Dogger Lake in the north (13). Current estimates of the maximum extent of glacial advances suggest a southernmost ice front that crossed Doggerland from northern Denmark to the Humber estuary of the United Kingdom 25.8 to 24.6 ka ago, leaving much of the eastern United Kingdom and southern Doggerland ice free (14), Fig. 1. By 18 ka ago the ice front had retreated to the latitudes of the Scottish borderlands. The extent to which there were heterogenous environments in these hinterlands capable of supporting temperate taxa remains an open question that has attracted increased attention in recent years (15–18).

## The Southern River System in Doggerland

**Fig. 2.**
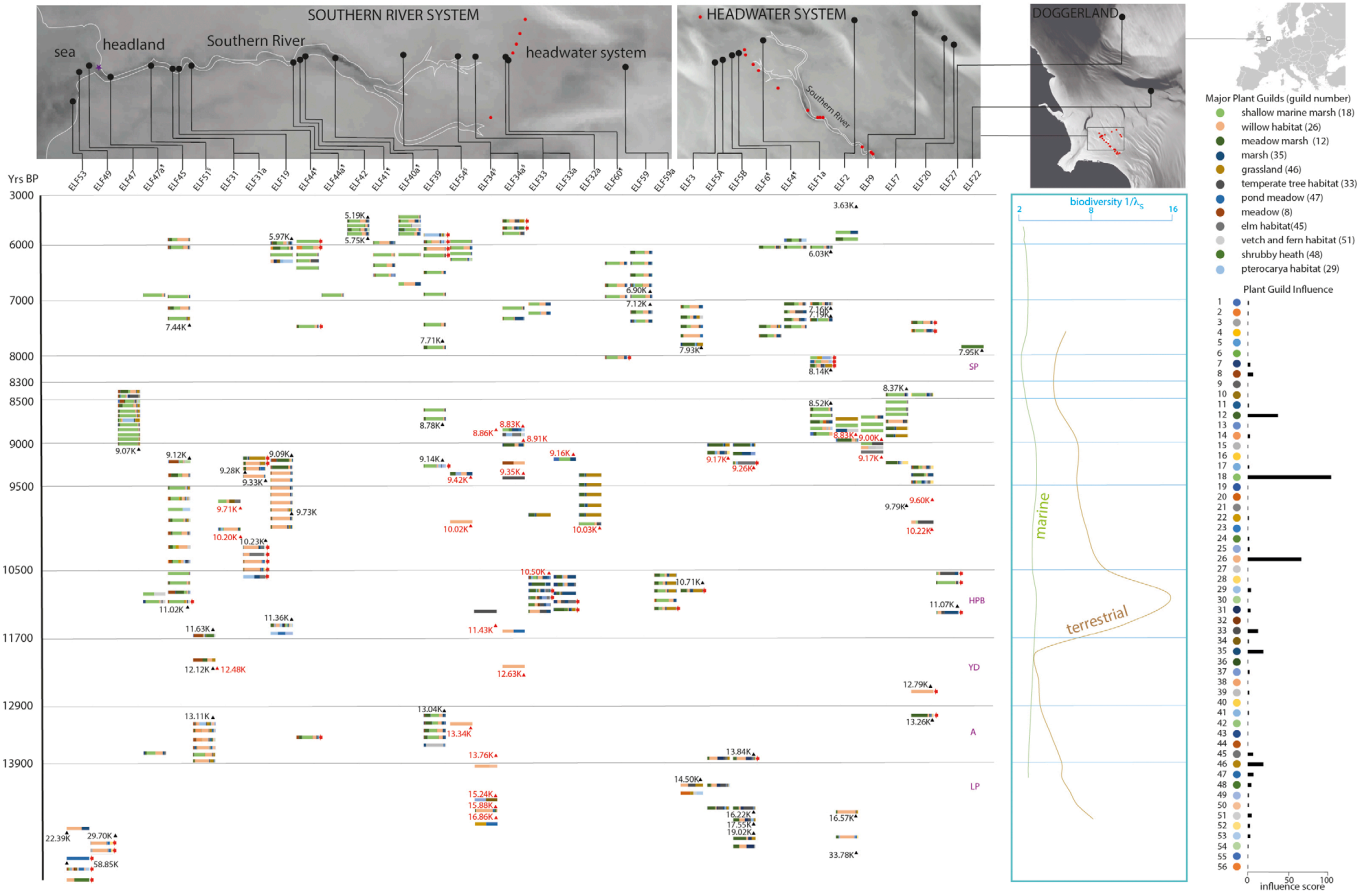
Plant guild profiles of the Southern River and Surrounding Doggerland area. *Top* panels indicate the location of the Southern River in Doggerland east of the British coast. Reconstructed Southern River system shown in white outline. Black dots indicate core locations, red dots indicate core sites shown in neighboring panels. The purple star indicates the site a hammer flint lithic was found (20). Plant guild profiles in which each color indicates a phytoassociative group of plants for each core are shown to the right with guild influence indicating prevalence. In order to drill down into specific bar graphs: guild order is preserved from left to right in bar profiles. Specific guild proportions for core samples are given in Dataset S8, taxonomic compositions of guilds are given in *SI Appendix,* Table S1, taxa counts for each core sample are given in Datasets S1 and S2. The 12 most common ecosystem types as indicated by plant guilds are shown top right (guilds shown in brackets). Red triangles indicate AMS dates, black triangles indicate OSL dates. § indicates cores subject to radiocarbon date age model profiles. indicates undated cores correlated with dated cores on the basis of profile similarity (see text). Red arrows indicate insecure sediments likely to contain reworked sediment signals (see text). Time periods are labeled LP (Late Pleistocene), A (Allerød), YD (Younger Dryas), HPB (Holocene Pleistocene Boundary), SP (Storegga Period). Biodiversity is shown as the reciprocal of Simpson’s λ_s_ for *Zostera* (marine) and other terrestrial dominated profiles. Note the time axis is scaled for sample density.

To understand the late glacial and early Holocene environment of Southern Doggerland, the Europe’s Lost Frontiers (ELF) team undertook seismic surveys and recovery of marine sediment samples for environmental and geochemical analysis between 2016 and 2019. Topographical reconstruction of the landscape characterized a river system, the Southern River (19), suitable to track the environmental changes that occurred from the Late Pleistocene to the period of inundation, Fig. 2.

The Southern River is around 30 km in length with a headwater area to the north close to the limit of the southern-most ice advance (Fig. 1). The river system opened into an estuarine mouth in the south between headlands which could have been suitable for human occupation, with likely plentiful freshwater and marine resources available to inhabitants (19). Recently, on the basis of this topography speculative dredging of the sea floor was undertaken in the estuarine mouth area leading to the discovery of a worked lithic from the early Holocene (20). Consequently, the Southern River is an area of particular interest with respect to its potential to support early Mesolithic occupation.

The goal of this study is to reconstruct the paleoenvironment of the Southern River system from the Late Glacial Period to the Holocene to investigate its potential to support Mesolithic peoples and track the process of marine inundation using sedimentary ancient DNA (sedaDNA). SedaDNA has proven to have an unprecedented resolving power of taxonomic reconstruction in Quaternary science (21, 22) and has the capacity to report highly localized signals (23–26) facilitating high-resolution paleoenvironmental reconstruction. To this end we present sedaDNA profiles obtained from 252 sediment samples obtained from 41 marine cores taken along a transect of the Southern River and across the headwater area toward Dogger Bank. By integrating sedimentological and sedaDNA data, we distinguish secure samples in which sedaDNA derives predominantly from a local source from those in which the signal derives from influxed and reworked sediments. Secure taxonomic profiles show the transition of the landscape from one supporting temperate trees from before a time contemporaneous to the Allerød, to an inundated environment dominated by sea grass (*Zostera*). The presence of thermophilous tree species too early and too far north given tree migration rates to have originated from classic glacial refugia, signal the existence of closer refugia in North-West Europe across glacial advances. We further detected a tree taxon now extinct in North-Western Europe, *Pterocarya*, in secure sediments and known to have occurred in the same area during previous interglacials. These results indicate the presence of an environment in the southern Doggerland area capable of supporting Mesolithic peoples from Pleniglacial times prior to the earliest known Maglemosian cultures (8).

## Results

### Paleoecological Reconstruction of the Southern River.

We initially carried out a broad scan of 239 samples across 41 cores of shotgun sequenced sedaDNA using the Illumina NextSeq 500 platform generating 1,071,136,936 reads. We used a triple database reference strategy to build ecological profiles, authenticate specific taxa, and resolve uncertain phylogeny respectively. First, we scanned the taxonomically broadest database available (Nucleotide NCBI) to avoid taxonomic ascertainment bias with a view to building ecological profiles of samples and filtered results with the Phylogenetic Intersection Analysis (PIA) algorithm which is remarkably robust to database gaps and inaccuracies (27) to recover high information value reads. Second, to increase read recovery of specific taxa, we drilled down into species of interest using individual reference genomes to establish individual DNA damage patterns (*SI Appendix,* Fig. S1). Third, we leveraged the WGS (NCBI) database to resolve uncertain phylogeny. In the first scan, we assigned reads with 96% accuracy to taxa within the *Viridiplantae* (Dataset S1) and Metazoa (Dataset S3) (27). On the basis of promising detailed taxonomic profiles spanning wide time ranges, a further 107 samples were selected across 9 cores (ELF7, ELF19, ELF20, ELF34A, ELF39, ELF44, ELF45, ELF47, and ELF51) including new sampling points to sequence to a greater depth, Dataset S2 (*Viridiplantae*) and Dataset S4 (Metazoa), generating a further 3,629,532,745 reads. In all 252 sampling points were taken and 4.7 billion reads were generated of which 0.1% passed through PIA filtering (27). We observed a 1,000-fold more reads assigned to plants than animals which we infer to broadly reflect their relative biomass presences in the environment, although it should be noted that genome size variation is particularly large in angiosperms (28) which biases their representation by DNA. We authenticated the sedaDNA as ancient by establishing a general signature of damage consistent with ancient DNA for Embryophyta and Metazoa respectively in this dataset (*SI Appendix,* Fig. S1 and Dataset S5) using MetaDamage (9).

To compare the ecological profiles of samples, we selected a subset of 445 European plant taxonomic assignations that were of a useful taxonomic rank, including 4 class, 39 order, 153 family, 80 tribe, 147 genus, and 10 of nonconventional rank and phylogenetically assigned with a *P* value of less than 0.0001, Dataset S6. We then sought to pull out high-level ecological patterns by identifying groups of plants that significantly co-occur with each other across samples (*Materials and Methods*, Dataset S7), which we term plant guilds (*SI Appendix,* Table S1). We identified 56 plant guilds and tested their robustness by assessing correlation enrichment within groups and used general linearized models to determine *p* values for groups’ nonrandom co-occurrence (*SI Appendix,* Fig. S2). All guilds were highly significantly enriched in correlations above the expected false discovery rate at the 5% and 1% levels, *SI Appendix,* Table S1. The majority of guilds (n = 48) were simple and reciprocal, with all pairs of plants in the guild significantly associated with each other. A minority of guilds (n = 8) were complex in that different groups of plants were associated with a specific anchor taxon. Plant guilds represent a combination of factors that result in depositional co-occurrence of taxa, including similar habitat and proximal location. Consequently, many of the guilds are characteristic of certain habitat types such as grasslands, meadows, or woodlands, so giving a high-level ecological signal. We found that guilds are also informative by their associations. For instance, the co-occurrence of *Hedera* (ivy) and *Ulmus* (elm) in Guild 45, combined with Arialiaceae (ivy family) and Ulmaceae (elm family) in Guild 24 to the exclusion of other tree species is suggestive of a prevalent ivy–elm relationship.

We used the plant guild system to build ecological profiles by assessing the proportion of guild membership for each sample, Fig. 2 and Dataset S8. We also identified the dominant ecologies by assessing the influence of each guild across the whole dataset summing up the proportions attributed to each guild across all samples. Four prominent ecosystem types are evident across our samples. The first is a shallow marine environment dominated by Guild 18 associated with *Zostera* (seagrass), evident throughout cores ELF47 and ELF45 near the mouth of the Southern River and in the upper strata of the majority of cores post inundation. The second ecosystem is willow habitat dominated by Guild 26 generally evident in lower strata of cores and particularly prominent in cores ELF51, ELF31A, and ELF19 representing the majority of the landscape prior to inundation. The third ecosystem is grassland dominated by Guild 46, often in combination with Guild 12 (meadow/marsh) seen in ELF32A, ELF7, and the upper section of ELF31A occurring in the upper reaches of the Southern River and headwater area prior to inundation. Finally, the fourth ecology is marsh environment dominated by Guild 35 usually in the presence of Guild 26 (willow habitat) and Guild 12 (meadow/marsh), evident in cores ELF5A, ELF5B, ELF33, and ELF33A occurring in the water catchment of the headwater area of the Southern River.

Overall, the ecological profiles show a coherent landscape of change from willow dominated woodland to a shallow marine ecosystem with inundation over time. Proximal samples showed a low ecological difference (∂; Dataset S9), indicating ecological consistency and a gradual rate of change. Adjacent cores are similar in many cases (mean adjacent sample ∂ = 0.349 SD = 0.379, mean within core ∂ = 0.456 SD = 0.414, mean between cores ∂ = 0.574, SD = 1.29 × 10^−8^), providing a possible basis for core alignment (such as between cores ELF5A and ELF5B). However, similarity between more geographically distant cores that are atypical of other samples within their own cores, such as in the case between cores ELF32A and ELF31A (∂ = 0.005) indicates the possibility of sediment reworking, highlighting the need to establish a robust taphonomy framework.

### Establishment of Secure sedaDNA Taphonomy.

Understanding the depositional and postdepositional processes for sedaDNA is crucial for interpretation of past environments (29), but formal methods to test these processes still need to be developed. We considered two aspects of taphonomy. First, postdepositional movement in which sedaDNA may have moved after incorporation into sediments through processes such as leaching. Second, predepositional processes considering how sedaDNA arrived at the point of deposition.

Postdepositional movement of sedaDNA is generally unexpected in marine sediments where there is low porosity and permeability, but might still be considered as a possibility (30). In the ELF cores, consistent Uranium/Thorium ratios down-core suggest a “closed system,” with limited exchange of radionuclides that might imply physical or chemical processes such as dissolution, sorption, and precipitation were active (31). The distance between sampling points ranged from 2.5 to 145 cm (mean 32.25 cm SD 23.76). We first tested whether read counts of taxa between adjacent core samples were significantly different to discount possible homogenization of samples. We found 86% of sample pairings met this criterion leading us to describe these as stratified samples (Dataset S10). We then estimated the proportion of read counts in one sample that could be explained by diffusion from an adjacent core sample under the assumption that DNA of different taxa would diffuse at the same rate (Dataset S11). The vast majority of samples gave maximum possible contributions to read count by diffusion at just a few or less than 1% (73% of samples < 5%, 69% samples < 3%), suggesting diffusion is not an influential postdepositional process in this dataset. However, in 14% and 16% of cases a nonstratified signal or diffusion respectively could not be ruled out. Given the overwhelming evidence of no postdepositional movement in other samples of similar sediment types in this dataset, we interpret these to be instances of stable environments in which taxonomic composition changed little between sampling time points.

To formally investigate the predepositional processes involved, we integrated sedaDNA and sedimentological data (*SI Appendix,* Fig. S3) into a sediment influx depositional model (*Materials and Methods*), *SI Appendix,* Fig. S4. The model accounts for the processes that contribute to the observed change (∂) in ecological profiles between adjacent samples in terms of the relative contributions from local sources in the immediate vicinity of deposition, and distal sources associated with influxing sediment. The model has been tested on a range of datasets demonstrating, for instance, that lacustrine sediments contain sedaDNA influxed from the wider catchment area (32). Across all sediments in our dataset, we estimate that 96% of sedaDNA comes from local sources rather than with influxing sediments in agreement with previous interpretations (23–26) (*SI Appendix,* Table S2). Importantly, we found a large difference between fine sediment types, such as silts and clays, and coarser sand sediments. In fine sediments local sources account for 89-98% of sedaDNA input, while the influence of influxing sediments associated with sedaDNA increases progressively in fine, medium and coarse sands reaching up to 70% of sedaDNA attributable to influxing. We also observe that laminated sediments are more associated with local sources than unstructured sediments. Fine sedimentation regimes represent slow moving water or low kinetic energy environments, whereas increasing grain size is associated with higher kinetic energy. These results indicate that only under conditions of rapid sediment influx is there also significant influx of environmental DNA from nonlocal sources. These results are congruent with current knowledge of environmental DNA (eDNA), which has a half-life of less than an hour in freshwater (33).

We interpret this pattern to mean that sedaDNA generally does not persist in influential quantities relative to local deposition through postdepositional sediment reworking processes in low kinetic energy environments. Consequently, we expect sedaDNA to track luminescence-depth profiles where these indicate coherent, unmodified stratigraphies, slow accumulation rates, and stable time-depth, since reworking also serves to reset optically stimulated luminescence (OSL) based ages (30). The taphonomy of tissues dated by accelerator mass spectrometry (AMS) dating might be less secure since these can persist through reworking episodes (9). However, high kinetic energy environments may deliver reworked sediments sufficiently quickly for redeposition with sedaDNA still preserved. The sedaDNA recovered from these sediment types therefore is more likely to represent older reworked sediments. This appears to be the case for ELF31A-58 which is from a coarse sand sediment reflecting closely the grassland profiles present in the older laminated silts of core ELF32A. We therefore conclude that unstructured sand sediments are prone to mixed ecosystem signals.

While the data here are sufficient to resolve differences in broad sediment types, larger sample sizes would be required to resolve individual sediment types within those categories. On this basis, we identified secure and insecure sediment samples where secure sediments reflect eDNA deposition contemporaneous to the local environment and congruent with OSL dates, Fig. 2. We based our further inferences of environmental reconstruction solely on secure sediments.

### Early Temperate Tree Colonization and Late Completion of Inundation.

In order to interpret the taphonomically secure ecological profiles in the context of the Pleniglacial and Holocene, we established a chronology through a comprehensive dating program of OSL (30) and AMS radiocarbon (34). In all 178 AMS dates and 139 quartz OSL dates were obtained across 16 and 22 cores, respectively, both of which were used to construct calibrated stratigraphies, Dataset S12. We used these date profiles to position samples on the age datum profile, Fig. 2. A remaining 8 cores did not have suitable material for either AMS or OSL dating. In these cases, we aligned samples to dated samples nearby closest matching by ecological distance (∂), *SI Appendix,* Table S15.

The ecological profile of the Southern River system begins in the Late Pleniglacial (35) over 20 ka (ELF53-214) and spans to the late Holocene 5.19 ± 0.17 ka (ELF42-60). Terrestrial biodiversity over this time period shows a steady decline in the Late Pleniglacial followed by an expansion of biodiversity that peaked 11 ka ago and declined rapidly thereafter, Fig. 2. A second decline occurred after 9 ka, possibly associated with rising sea levels, previously also observed on the Irish Sea coast (36). The general profile of terrestrial biodiversity change matches closely to that of the Circum-Arctic region (37) suggesting that although this is a small specific local analysis rather than a wide regional one, it may reflect a general northern hemisphere trend.

**Fig. 3.**
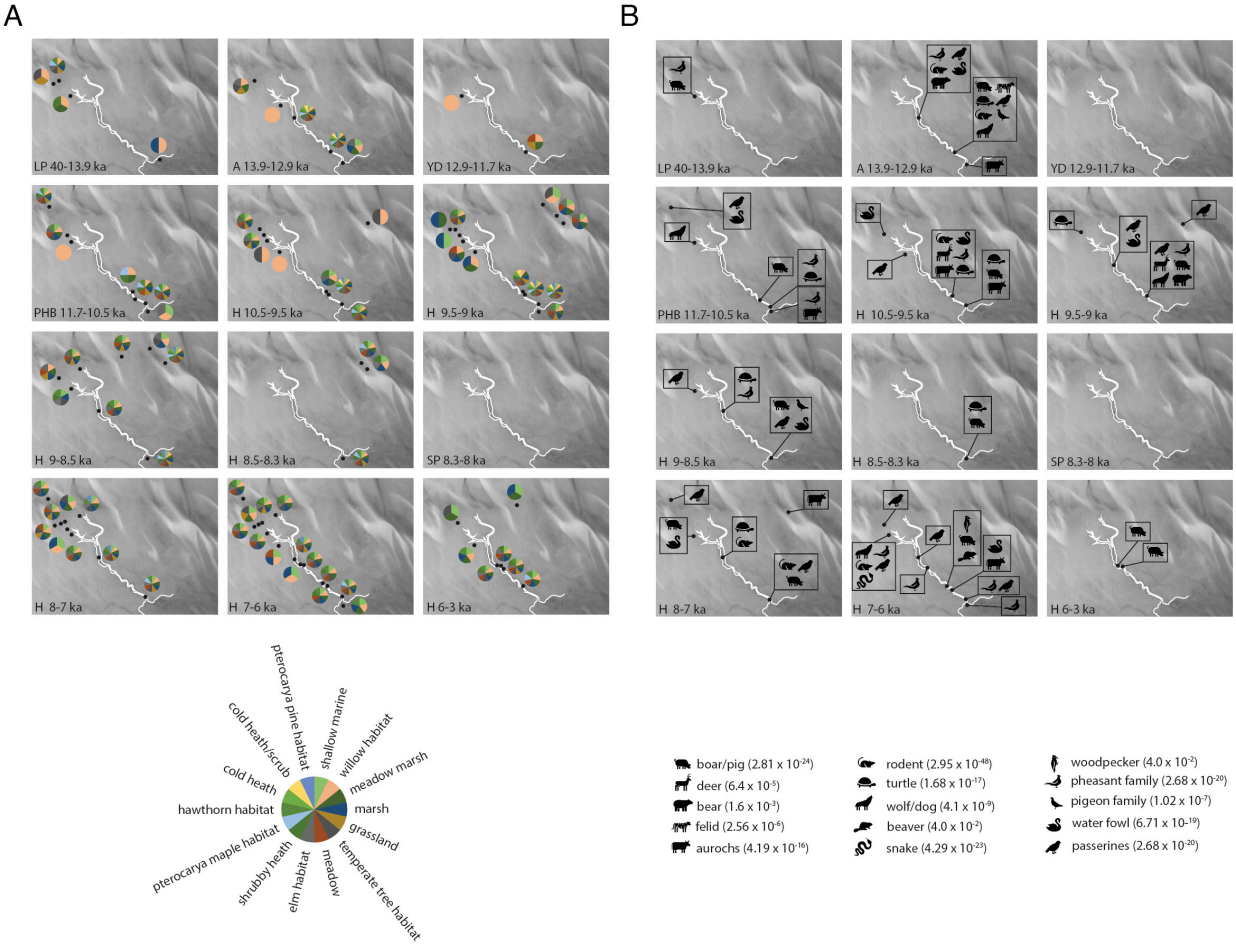
Major plant guild and animal occurrence over time in the Southern River. Reconstructed Southern River system shown in white outline for time periods indicated in Fig. 1. Black dots indicate core locations. (*A*) Pie charts indicate presence only of the 14 most influential plant guilds displayed clockwise in order of influence: shallow marine (guild 18), willow habitat (guild 26), meadow marsh (guild 12), marsh (guild 35), grassland (guild 46), temperate tree habitat (guild 33), meadow (guild 8), elm habitat (guild 45), shrubby heath (guild 48), pterocarya maple habitat (guild 29), hawthorn habitat (guild 24), cold heath (guild 6), cold heath scrub (guild 28), pterocarya pine habitat (guild 13). (*B*) Icons indicate presence only of 15 major animal groups detected, phylogenetic assignation *P* values shown in brackets.

**Fig. 4.**
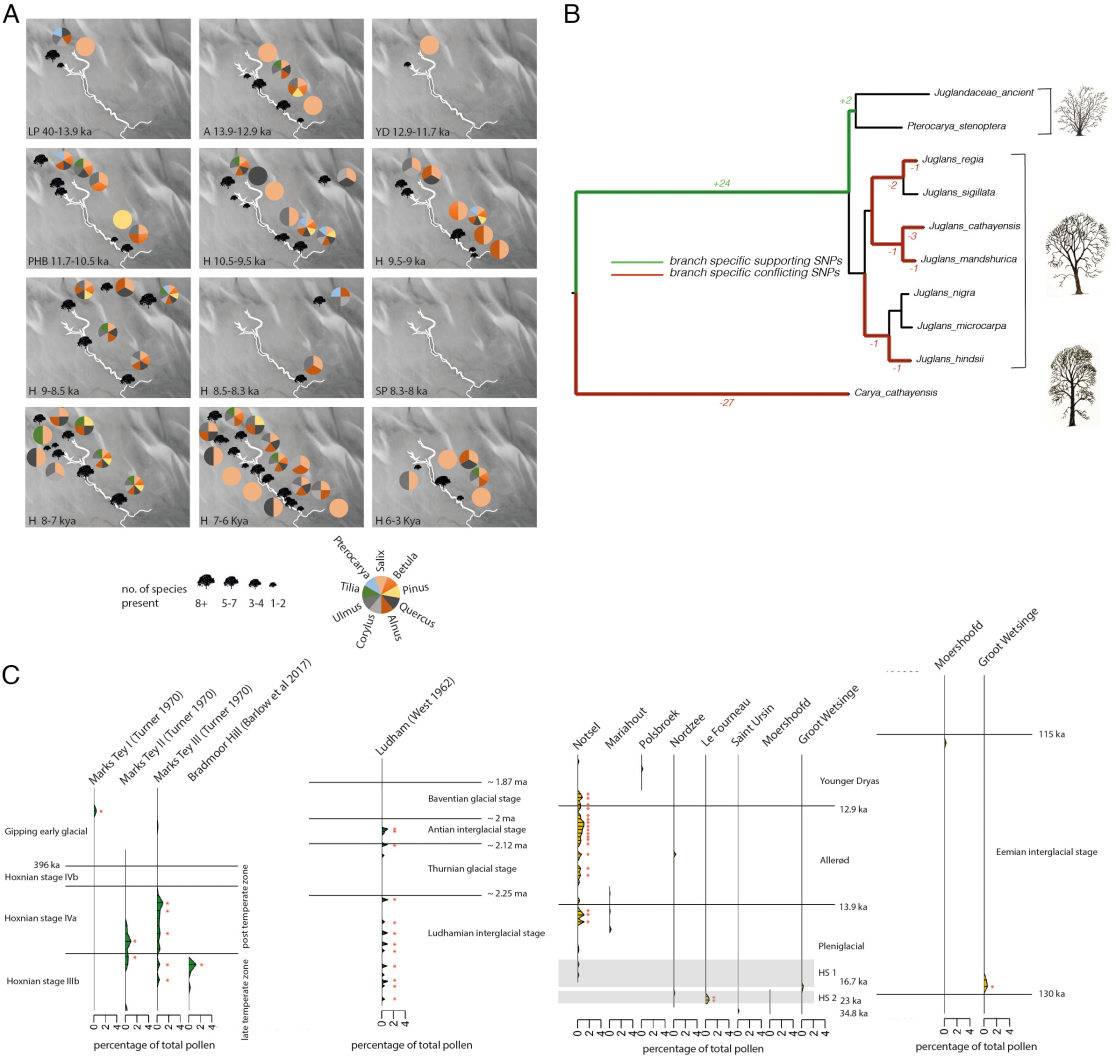
Major tree species occurrence over time in the Southern River. (*A*) Pie charts indicate presence only of nine major tree species. (*B*) Phylogenetic reconstruction of ancient Juglandaceae. Tree constructed using raxml-ng (39). Green branches indicate SNPs supporting those particular branches for a Pterocarya/ancient Juglandaceae clade, red branches indicate SNPs which conflict with a placement with ancient Juglandaceae. (*C*) Pollen profiles of established *Pterocarya* (green) from Ludham, Marks Tey, and Bradmoor Hill and anomalous *Pterocarya* (yellow) from Notsel, Mariahout, Polsbroek, Nordzee, Le Fourneau, Saint Ursin, Moershoofd, and Groot Wetsinge. HS: Heinrich Stadial event. Samples in which *Pterocarya* meets the standard threshold (40) of 0.5% of total pollen are shown with red asterisks.

Surprisingly, over 16 ka prior to the Allerød warm period we found temperate tree habitat inhabited by boar in the upper headwater area of the Southern River (Fig. 3 *A* and *B* and Dataset S13), including the genera *Quercus* (oak), *Alnus* (alder), *Corylus* (hazel), and *Ulmus* (elm), Fig. 4*A* and *SI Appendix,* Figs. S1 and S5. While these tree taxa have been detected at trace levels by pollen at 15 ka, it is not until 10.5 to 9.5 ka that they have been detected at high frequencies on the British mainland (38). Given the presence of a forest dwelling faunal species, this result suggests a substantial and human exploitable environment indicating a very early post glacial colonization by trees in this area.

The Allerød (13.9 to 12.9 ka) sees biodiversity expand to include *Tilia* and animal species in the mid reaches of the Southern River including bear and European terrapin, fossil evidence of which has previously been detected in Denmark at 10.6 to 9.1 ka and reached southern Sweden by 9.8 ka (41, 42). Only three of our samples date to the Younger Dryas (12.9 to 11.7 ka) and only *Salix* (willow) is detected at this time. Subsequent to the Younger Dryas, most species reappear prior to 10.5 ka. This is 2 ka before *Tilia* and 1 ka before *Corylus* become common on the British mainland respectively (38).

In the lower reaches of the Southern River system (ELF51, ELF31, ELF31A, and ELF19) the preinundated landscape of the early Holocene (10.5 to 9 ka) supported willow habitat, which became marshy toward the end of that period. Contemporaneously, the upper reaches of the system (ELF32A, ELF7, and ELF33) were dominated by marshy grassland that also supported trees. During the early Holocene (9 to 8.5 ka) the onset of inundation generally becomes apparent with the switch to *Zostera* dominated marine environments (ELF39, ELF1A, ELF2, ELF7, and ELF9). We previously reported evidence of the Storegga tsunami at 8.15 ka in ELF1A (9). Unsurprisingly, the samples from this time period are insecure for sedaDNA as great swathes of the landscape were torn up and redeposited giving a mixture of influxed sediment and a woodland signal from deposition of plant material. However, the persistent terrestrial signal observed previously in ELF1A posttsunami is also observed in secure sediments across the landscape. This pattern suggests that land was still close enough for direct local deposition into the Late Holocene, the effects of inundation were heterogeneous, and final submergence possibly as late as the 6th millennium BP.

We considered our results in the context of other environmental proxies that have been taken from these cores (43). Pollen and macrofossils have been recovered from six cores where preservation was appropriate spanning a time period from the Younger Dyas to 8.3 ka. For this period, we see complete congruence between sedaDNA, pollen, and macrofossils (Dataset S14). However, the sedaDNA record in this case spans a greater number of sediment types giving a more complete paleoecological record over time.

### Identification of a Locally Extinct Taxon: *Pterocarya*.

We were surprised to find a substantial signal for Juglandaceae (walnut family) from the late Pleistocene and peaking in the early Holocene, Fig. 4*A* and *SI Appendix,* Fig. S4. The nearest European species, *Juglans regia* (English walnut), is not detected in the palynological record in northern Europe until 8.5 ka (38). Juglandaceae has appeared in several sedaDNA studies as an unexpected exotic (24, 25), including in conjunction with pollen (24). Although control blanks were clear, we further ruled out contamination as a likely source by establishing a clear ancient DNA damage profile associated with Juglandaceae that closely matched that of other recovered tree species, *SI Appendix,* Fig. S1. To investigate the identity of this taxon further we aligned 206,113 bases of sedaDNA from 4,904 reads assigned to the Juglandaceae against whole genome data from 8 Juglandaceae species, Dataset S16, and carried out phylogenetic analysis (*Material and Methods*). The sedaDNA of the ancient Juglandaceae robustly forms a clade with *Pterocarya stenoptera* leading us to conclude that this represents a member of the *Pterocarya* genus, Fig. 4*B*.

*Pterocarya* is a relict riparian genus that used to be widespread in distribution but is now extinct in North-Western Europe (44, 45), consequently its presence in this dataset is surprising, and we considered pollen records as a possible source of supporting evidence. *Pterocarya* leaves relatively little pollen in the palynological record, typically sporadically at levels of around 0.5 to 1% of total pollen, making it difficult to establish presence (Fig. 4*C*). It was originally thought that the genus went extinct in northern Europe after the Antian interglacial stage 2 Mya (46), but later evidence from Marks Tey (47) in the South East UK indicated a presence in the late and post temperate phases of the Hoxnian (426 to 396 ka). Accepted *Pterocarya* pollen has also more recently been recovered from Bradmoor Hill, a Hoxnian aged site nearby the study area (48). However, there are numerous instances of anomalous *Pterocarya* reported in the European Pollen Database (49). We found *Pterocarya* reported in all cores spanning 200 to 100 ka and 21% of cores spanning 50 to 10 ka, representing 47 separate samples across 8 cores suggesting a pervasive presence in this geographical area (Fig. 4*C* and Dataset S15). These anomalous instances of *Pterocarya* are mostly localized to warm periods in the Eemian, Allerød, and the last two Heinrich Stadial warming events of the Pleniglacial in a climatic profile which closely matches previous accepted instances of *Pterocarya*. Application of strict standard threshold pollen frequencies used to discount reworked pollen (40) indicate as much support for presence of *Pterocarya* in the anomalous pollen as with the older accepted pollen profiles of the Hoxnian and Antian. Even where *Pterocarya* is present, the evidence from pollen is so marginal that an appropriate level of caution should be applied. Nonetheless, palynological evidence for Late Pleistocene *Pterocarya* meets any authenticity standards that could be applicable to the earlier Hoxnian deposits.

It is possible that some of the reworked sediments in the ELF cores could originate from Hoxnian time periods and explain the presence of a *Pterocarya* sedaDNA signal. However, there are large problems with an inference of a Hoxnian aged signal. Although ancient DNA has been recovered from samples on this timescale, they have almost exclusively been recovered from stable permafrost conditions (21, 22, 50, 51). There have also been some unusual instances reported of mid Pleistocene aged DNA at more comparable latitudes to this study in highly stable cave systems, with the DNA in these rare cases being exceptionally degraded (52, 53). The preservation of DNA is linked to low temperatures and importantly, temperature stability (54). However, our study area does not represent a stable environment over this time frame and prior to inundation cycled through numerous warming episodes as an exposed terrestrial system making persistence of DNA unlikely. Where ancient DNA has been reported of this age, the frequency of observable cytosine deamination mismatch signal indicates saturation (22), which generally occurs in the 40 to 50% range of first position C->T misincorporations. Our datasets are substantially deaminated as expected, but well short of the saturated deamination previously observed for middle Pleistocene aged DNA even from stable conditions. Therefore Hoxnian aged DNA would be clearly distinguishable from Late Pleniglacial aged DNA in this system. The *Pterocarya* deamination signal we observe is close to 30%, and indistinguishable from the signals we recovered from *Quercus*, *Corylus*, *Salix*, *Alnus,* and *Ulmus*. There is therefore no indication that this is a significantly older signal. Finally, *Pterocarya* was identified in secure sediments (ELF19, ELF45) and unlike many other species is largely absent from sediments likely to carry a reworked sedaDNA signal. We therefore conclude a genuine signal of relict *Pterocarya* in the Late Pleniglacial and infer that its final extinction in North-West Europe was later than has previously been recognized.

## Discussion

### An Emerging Picture of Northern Refugia in the Early Mesolithic.

Our results suggest a climate in the Doggerland area that supported temperate trees over 16 ka—into the last glaciation—in contrast to the expectations from the pollen records of the surrounding European uplands (38). However, very little palynological work has been carried out in Doggerland to date, but where it has the records agree with our findings. The work we present here suggests that Pleniglacial temperate tree pollen will likely be found in the future if suitable preservation conditions can be located. The contrasting taphonomies of sedaDNA and pollen are evident in the fact that they are preferentially preserved in different environments, in part driven by the variances in their chemical vulnerabilities and modes of dispersal. The durable nature of sporopollenin (55) facilitates its occurrence in sediments such as peat where DNA is typically prone to poor preservation because of acid hydrolysis processes (56). Conversely, we find sedaDNA accumulates well in alluvial deposits but does not persist well through slow reworking episodes. In these environments pollen can be more sporadic in occurrence and persists through reworking potentially making interpretation complex. It has long been recognized that pollen is unlikely to be an influential source of sedaDNA (21). Unlike pollen, DNA does not have a known aerial route for dispersal at scale. Because of these contrasting taphonomies, sedaDNA and pollen can complement each other either in differential preservation in different sediment types, or where both occur in fine sediments by providing a local and regional signal respectively (9). Finally, our sediment influx depositional model suggests that in lacustrine systems both sedaDNA and pollen may give complementary regional signals.

There has been increasing speculation in recent years on the existence of cryptic or microrefugia to explain the increasingly apparent early colonization of biota into Northern Europe (15–18, 57–59). This early colonization is termed Reid’s Paradox, because the dispersal rates from classic glacial refugia required for tree species appear to exceed tree dispersal rates (60). The evidence indicating early colonization within the Southern River System accentuates this paradox further but also suggests a solution. The early dates we observe imply colonization of Britain and perhaps mainland Europe from the Doggerland area in the Holocene, and the retention of multiple thermophilic species of flora and fauna from the closing stages of the Late Pleistocene and Allerød periods through the Younger Dryas. The Southern River flowed through a glacial tunnel valley (9), which could plausibly have provided a microrefugium as has been suggested for other valley systems (17, 61, 62). Equally, such a microrefugium could well have been located somewhere else in the landscape within a radius compatible with such early colonization. Niche modeling has predicted the possible survival of temperate summer green deciduous tree taxa through the glacial maximum in this area based on broad regional trends (63). Furthermore, recent advances in the effects of modern microclimates show that landscape heterogeneity leads to a general underestimation of suitable habitat area at a regional scale (64), making it increasingly likely that northern refugia could have existed within an otherwise unpromising region.

A wooded ecosystem capable of supporting forest fauna such as boar prior to the Allerød suggests a human exploitable landscape at around 16 ka, substantially predating the earliest known Maglemosian culture (8). This is a time associated with the breakdown of Upper Paleolithic cultures with megafaunal extinction and switch to the exploitation of forest-tundra species and aquatic resources (65). Paleogenomics indicate that peoples in this region would have been of Fournol ancestry giving rise to Magdalenian culture (66) and of their descendents, members of the early Mesolithic Ahrensburgian culture associated with reindeer occurred in this region 12.9 to 11.7 ka (65). Given the need to be near the coast during this transitionary period to exploit aquatic resources it is likely that peoples would have been drawn into the southern Doggerland landscape during the late Pleniglacial period. The hammerstone recently recovered from the mouth of the Southern River cannot be ascribed to a specific Upper Paleolithic or Mesolithic culture (20), but is the first example of a targeted attempt to recover archaeological artifacts from the Doggerland area rather than a chance find. Further targeted prospecting may be expected to unearth more derived lithics characteristic of the Ahrensburg or Magdalenian on the basis of the results we present here. Notably, recent evidence shows Late Glacial Ahrensburgian occupation of western Scotland supporting our findings (67).

A wide range of analyses are ongoing on the Southern River by the Europe Lost Frontiers team which will further refine our understanding of this landscape in the future in terms of geomorphology and environment (68). The findings presented here raise the possibility that northern refugia enabled the early colonization of Doggerland facilitating an environment in which the Mesolithic could evolve from the Upper Paleolithic. The resolving power of sedaDNA is evident in the strong evidence for the presence of a regionally extinct taxon, *Pterocarya*, which can only be marginally established through pollen in this region. This further indicates the role northern refugia may have played through the last few glacial cycles and is an area worthy of further investigation.

## Materials and Methods

### Sampling.

Details of the coring from the European Lost Frontiers program have been published elsewhere (69). Coring was undertaken in two stages. Cores 1 to 20 were taken in 2016. Cores 21 to 60 were taken the following year. Cores 1 to 20 were taken with 5 m vibrocorers to collect continuous 86 mm diameter samples. Cores 21 to 60 were taken with 6 m vibrocores and 86 mm diameter samples. Opaque liners were used to allow (OSL) dating. The cores were sealed and wrapped in black plastic immediately upon recovery. The sealed cores were then transported to dedicated aDNA facilities at the University of Warwick where sampling for DNA was the first process undertaken.

### sedaDNA: Data Generation.

#### DNA extraction.

All DNA handling stages prior to PCR took place in a dedicated aDNA facility at the University of Warwick following standard protocols for processing ancient DNA (70). Sealed sediment cores were refrigerated at 4 °C immediately after retrieval and were held at a constant 4 °C until sampling. Cores were split under strict aDNA lab conditions and under red-light to preserve samples for OSL analysis. All samples for aDNA work were taken inside a category two biosafety cabinet using sterile equipment. Sampling points were guided by sedimentological patterns to ensure different sediment types were included. The cut surface of the core was removed and ~20 g of sediment retrieved, ensuring that no sediment from the outer 1 cm of the core was collected, as this may have been disturbed during the coring process. For DNA extraction, library preparation, sequencing, and downstream analysis, each sample was processed in duplicate. For each duplicate, 2 g (±0.05 g) of sediment was taken from each sample. Subsamples were processed in batches of up to seven plus one negative control (reagents only). The subsamples were mixed with 5 mL CTAB buffer (2% w/v CTAB, 1% w/v PVP, 0.1 M Tris pH 8.0, 20 mM EDTA, 1.4 M NaCl) and incubated at 37 °C with agitation for 7 d. After incubation, the subsamples were centrifuged at 20,000×*g* for 10 min. The supernatant was moved to a new 50 mL tube and manually shaken with 4 mL chloroform:isoamyl alcohol (24:1) for 5 min. The resulting mixture was centrifuged at 20,000×*g* for 5 min. The aqueous phase was combined with 20 mL Buffer AW1 (Qiagen) and incubated at room temperature for 1 h. This was then applied to silica-based spin columns using a vacuum manifold. The columns were then washed, first with 500 μL Buffer AW2 (Qiagen), and then 300 μL acetone, both followed by centrifugation at 6,000×*g* for 1 min. The columns were then removed from their collection tubes and air dried for 5 min. Finally, DNA was eluted in 65 or 75 μL Buffer EB (Qiagen). They were incubated at 37 °C for 10 min and centrifuged at 15,000×*g* for 2 min. The eluted DNA was quantified using a high-sensitivity Qubit assay (Invitrogen).

### Sequence Generation.

The library protocol is based on that of Meyer and Kircher (71) with the following modifications from Kircher et al. (72): 0.1 μL of adapter mix during adapter ligation instead of 1 μL; spin column purification (MinElute PCR purification kit, Qiagen) instead of SPRI; purification step after adapter fill-in replaced with heat inactivation for 20 min at 80 °C; Double indexing; no fragmentation step, as ancient DNA is expected to already be shorter than 400 bp; blunt-end repair reaction volume of 40 μL; T4 DNA ligase added to individual sample tubes instead of the master mix during adapter ligation; Platinum Pfx was used indexing PCR the initial screening of 239 samples (PIA outputs in Datasets S1 and S3), but since this was discontinued in 2018, Platinum SuperFi was used for deep sequencing the last 107 samples (PIA outputs in Datasets S2 and S4); there were 16 PCR cycles for samples in which Platinum Pfx was used, but where Platinum SuperFi was used 18 PCR cycles were applied.

Libraries were visualized on a 2% agarose gel. They were then cleaned using 45 μL SPRI beads and eluted in 20 μL TET buffer (73). The cleaned libraries were quantified using a Qubit assay (Invitrogen) and a fragment size profile produced using a Bioanalyzer (Agilent). Libraries were normalized to 4 nM and pooled prior to sequencing on the Illumina NextSeq 500 platform using the high-output, v2, 150-cycle kit (75 × 75 paired end). Sequence data were deposited in the NCBI SRA (project code PRJNA1001812).

### Bioinformatics.

Raw BCL files were converted to FASTQ and demultiplexed using Illumina’s bcl2fastq software (version v2.20.0.422), using the --no-lane-splitting and --ignore-missing-bcl options. Adapters were removed and paired end reads were collapsed using AdapterRemoval version 2.2.2 (74), specifying a minimum length of 30 and a minimum quality of 30. FastQC version 0.11.6 (75) was used to visually assess the success of adapter and quality trimming. FASTQ reads were converted into FASTA format using the following example shell command: In.fastq | awk ‘NR%4 !=0’ | awk ‘NR%3 ! = 0’ | sed ‘s/@/>/g’ > out.fasta-->. Finally, duplicates were removed using the fastx_collapser command from the FASTX-toolkit version 0.0.13 (Hannon, G.J. (2010) FASTX-Toolkit. (http://hannonlab.cshl.edu/fastx_toolkit).

An initial metagenomic BLASTn search version 2.6.0 (76) was undertaken using the tab output (specified using -outfmt “6 std staxids”) and otherwise default parameters of the Nucleotide (NCBI) database. This allows a large volume of data to be processed with a far smaller data footprint than the full BLAST output format. This was then converted to RMA format using the MEGAN command line version 5.11.3 (77), enabling the visualization of the preliminary data. The patchiness of DNA sequence databases, overrepresentation of model organisms and database errors leads to unreliable assignation of sequences. Reads were therefore stringently filtered using the Phylogenetic Intersection Analysis (PIA) using the 2019-09-01 NCBI taxdump release, which has been shown to have a 96% accuracy with this dataset (27) (https://github.com/Allaby-lab/PIA). Briefly, accuracy for this dataset was determined by subjecting to PIA taxonomically known sequences identified by BLASTn hits of sedaDNA, under the condition of database masking for each taxon (27). Default parameters were used in which threshold taxonomic diversity score was 0.01, equating to phylogenetic ranges being based on information from at least 6 different taxa, and a minimum coverage match of 95% of the length of a read. The false positive rate was determined as 1 – accuracy (1 − 0.96 = 0.04). We leveraged this false positive rate to assign *P* values to taxonomic assignations based on read count as *P* = (false positive rate)*^n^* where *n* equates to read count. FASTA sequence reads with preliminary assignation to taxa of interest (in this case Viridiplantae, Chordata with primate reads excluded, Arthropoda, and a random subset of 10,000 bacterial reads) were extracted from the RMA files using MEGAN5 command line tools version 5.11.3 These reads were subjected to a second round of BLASTn, this time to generate the full BLAST format as an output. These were then used as an input for PIA in order to retrieve stringent assignations. Taxa present at >2% of the sample read count in the negative control were removed. Any taxa that remained after the stringent filtering that were not native to Europe were discarded, accounting for about 3% of the data. We compared our genus level findings with the WGS (NCBI) database and found that 30% of the genera retrieved were absent from WGS affirming our strategic rationale to recover the widest possible range of taxa present by utilizing Nucleotide with a robust phylogenetic assignation approach.

### Damage Assessment.

Since the read count for any single taxon can be relatively low, we took the approach of assessing damage across all taxa of a particular group using MetaDamage (9). Samples were compared to the NCBI Nucleotide database. For individual species (*Quercus*, *Alnus*, *Tilia*, *Corylus*, *Salix,* and *Ulmus*) reads were subsampled from the BLAST dataset using MEGAN and mapped against their respective genomes (*Quercus*: GCF_932294415.1; *Alnus*: GCF_958979055.1; *Tilia* GCA_020138205.1; *Corylus*: GCF_901000735.1; *Salix*: GCA_027405865.1;*Ulmus*: GCA_010015005.3) with bwa-0.7.15 using the BWA-MEM algorithm (78). Mapped reads were then analyzed with MetaDamage. See section *Identification of Pterocarya* for details on treatment of Juglandaceae reads.

### Plant Guild Generation.

To reduce data complexity and make trends more discernible, we adopted a strategy based on using the covariance matrix of taxa to plot orthogonal axes in high dimensions to reveal covariance trends (79). We developed the approach of constructing plant guilds based on the notion of groups of taxa that tend to occur together in samples. To construct guilds of plants from read count data and compare samples we used a series of custom scripts to carry out the following manipulations. First we calculated the Pearson product moment correlation coefficient (pmcc) between each pair of taxa for their frequency in each sediment sample across all sediment samples that were deep sequenced. We used the pmcc values as a measure of similarity in co-occurrence between taxon pairs, then converted these values, ranging from −1 to 1 to distance values to make linear vectors between taxa pairs ranging between 0 and 1 using Eq. **1**:

[1] $$distance=1-\frac{pmcc+1}{2}.$$

We then generated coordinates for each taxon in 10 dimensions using a multidimensional scaling (MDS) plot in R from the distance matrix. Hypercubes were generated by dividing each dimensional axis into three equal sections and then designating cube coordinates by allocating one of the three sections from each of the dimensions under consideration. All taxa coordinates that occurred within the boundaries of a hypercube were then grouped together. Beginning with taxa grouped in three dimensions, we progressively split groups by introducing more dimensions until the group was considered a stable cluster, that is it was not split significantly by incrementing dimensions.

The dataset as a whole, agnostic to guilds, had a modal correlation of −0.009, indicating little correlation across all taxa. The guild method is expected to identify groups with enriched correlation, we therefore further evaluated guilds by examining within guild correlations. We found two guild types. The majority (46) were of the first type, simple, in which the mean correlation and SD range were substantially above zero, indicating a general coherence within the group of co-occurrence. A minority (8) were of the second type, complex, in which although the mean correlation was above zero, the SD range extended into the negative. On examination these groups are often driven by low count numbers of some taxa, and where different groups of taxa have been identified as associated with an “anchor” taxon, such as *Vaccinium* in guild 19 or vetch and fern groups in guild 51. Alternatively, ecologically similar groups often with similar assignations between the subgroups occur but at different classification ranks, as in the case of guild 49. Each of the complex guilds on examination is ecologically coherent, although of little influence in the analyses, all the major guilds on which influence the analyses are of the simple type. We then further evaluated the guilds with generalized linear models (GLM) based on the negative binomial distribution using the manyglm () function of the mvabund R package (80). Pairwise *P* values were generated to establish whether taxa were significantly correlated (*P* < 0.05) and their distributions plotted for each guild. We assessed whether correlations occurred in each guild with *P* values below 0.05 or 0.01 above the expected false discovery rate at the 5% and 1% levels using the Binomial distribution in which X ~ Bin(guild size, *P*), where X is the observed number of correlations below the 5% or 1% levels respectively and *P* is the false discovery rate. All guilds were significantly enriched for *P* values below significance thresholds above the false discovery rate, *SI Appendix,* Table S1. We also checked for bias effects of count number on guild composition but found no correlation.

Samples were then assigned a percentage guild membership based on the proportion of reads present belonging to taxa of a particular guild. A guild profile was then built up based on the percentage membership of all guilds present. The guild distance (∂) between samples was calculated as

[2] $$\partial =\sum_{1}^{N}(p1_{i}-p2_{i}{)}^{2},$$

where *p1* and *p2* are the *i*th guild proportions for samples 1 and 2, respectively, and N guilds are considered. Values of ∂ range between 0 and 2.

### Biodiversity Analysis.

To calculate biodiversity over time we used Simpson’s Index (81) calculating λ_s_ (denoted here as λ_s_ to distinguish it from the diffusion parameterλ) by summing the square of the proportions of each taxonomic read count in each sample. We then used 1/λ_s_ values to indicate diversity. Secure core (see determination in section *Taphonomic sediment influx analysis*) samples with total read counts over 50 and absolute dates (49 in total meeting the criteria) were ranked by age and separated into marine or terrestrial dominated samples. A thousand year sliding window with 500-y steps was used to calculate average diversity values for each time period.

### Stratification Analysis.

We established how stratified sample signals were by using an approach applied previously (9). Briefly, Beta distributions were used to explore the underlying distributions giving rise to observed read counts for individual taxa, given the total read count for each sample, where the a parameter was (1 + taxa count) and the b parameter was ([total read count – taxa read count] + 1) and the sum of read counts of the two samples was greater than 50. Probabilities that the read counts of a single taxon from the samples were drawn from the same underlying distribution were calculated from the overlap in the two respective beta distributions. We anticipated that for some taxa little environmental change would have happened between samples and hence would not be expected to be significantly different between samples. The presence of taxa that did significantly differ between samples was therefore taken as evidence of stratification. While most samples showed multiple significantly different taxa, we show the taxa with the most significant *P* values in *SI Appendix,* Table S10.

### Diffusion Analysis.

We explored the possibility that sedaDNA may have diffused between adjacent samples and influenced taxon read counts in cases where the total read count of the two samples was over 50. In this case differences between samples would be due to two counteracting components, first the differentiation in environment over time, and second, the homogenization of counts by diffusion. While pure diffusion is expected to follow Fick’s laws of diffusion, diffusion through a porous medium is non-Fickian and follows a degenerate parabolic shape (82). We applied a simple unidirectional approximation of a diffusion curve shape described by the parameter λ such that

[3] $$\lambda =\frac{-\log\left(\frac{p_{1}}{p_{2}}\right)}{x},$$

where *p1* and *p2* are the proportions of read counts attributed to a taxon in samples 1 and 2, respectively, and *x* is the distance in cm between the two sampling points. Lower values of λ describe shallower curves and therefore more complete diffusion processes. The value of λ that described the count of one sample being the result of diffusion only from the other was calculated for each taxon in the samples. We reasoned that a single average universal value of λ should apply to all sedaDNA between two samples that would be dependent on the sediment porosity rather than the DNA or the taxon from which it came. To identify the minimum possible value for λ we identified clusters of values from the frequency distribution of all taxa, and reasoned that the highest cluster represented the maximum diffusion rate possible in the sample, although it is possible that no diffusion occurred at all and this class simply represents the highest category of environmental change. We took the average of this cluster to represent a reasonable estimate of the minimum possible value for universalλ, although the true value could be much higher and effectively describe little or no diffusion. We then applied this value of λ to determine the expected read count of a taxon expected in sample 1 by diffusion from sample 2 (*C_diff_*):

[4] $$C_{\mathrm{diff}}=e^{-\lambda x}.p_{2}.s1_{\mathrm{total}},$$

where *s1_total_* is the total read counts in sample 1. The total number of read counts attributable to diffusion were summed up for all taxa and expressed as a percentage of the total number of read counts of a sample. This value was then taken as the maximum possible amount of diffusion explicable by the data, Dataset S11.

### Taphonomic Sediment Influx Depositional Model Analysis.

We considered two contrasting situations in which sediment type does or does not change between adjacent samples in a core, *SI Appendix,* Fig. S4. The contribution to the difference in guild structure between two adjacent samples of the same sediment type (∂1) can be described as the change in the sedaDNA associated with influxing sediment 1 (*d_xsed1_*), and the change in sedaDNA from the environment of more local sources (*d_xloc_*):

[5] $$\partial 1=\delta_{\mathrm{xloc}}+\delta_{xsed1}.$$

Both terms are concerned with environmental change. In contrast, the contribution to guild distance between two adjacent samples of different sediment types (∂2), involves *d_xloc_*and the change associated with the compositional difference between sediments 1 and 2 (*∂_xsed1-2_*):

[6] $$\partial 2=\delta_{\mathrm{xloc}}+\partial_{xsed1-2}.$$

The amount of change between sequential samples due to local sources will be a function of the compositional proportion of the sedaDNA that is of local origin (*cl*). We reasoned that the amount of change associated from the sedaDNA influxing with sediments would be determined by the compositional proportion of sedaDNA associated with sediment (*cs*), which must be equal to 1-*cl* if there are no other sources, modified by a parameter (*e1*) that accounts for the difference in the rate of environmental change associated with the sediment source area relative to the local area:

[7] $$\partial_{xsed1}=\frac{\delta_{\mathrm{xloc}}}{\mathrm{cl}}∙cs(1+e_{1}).$$

The compositional change between sediments 1 and 2 *(*∂*_xsed1-2_*) is expected to be a function of the plant guild score difference between the environments associated with sediments 1 and 2 (*id*), and the compositional contribution of the sediment. However, the change in the sedimentation regime is likely to be accompanied by a change in the sedimentation rate and so the overall contribution to change that influxing sediment makes, which we account for with a second environmental variable (*e2*):

[8] $$\partial_{xsed1-2}=id∙cs∙\left(1+e_{2}\right).$$

For each pair of environmental variables, *e1* and *e2*, there are just three independent variables: *cl*, *∂_xloc_* and *id*. To find the combinations of variables that best fit the observed values of ∂1 and ∂2 for each sediment type we used a Monte Carlo approach to search values of *e1* and *e2*, for each pair evaluating the closest fit from all values of *cl, ∂_xloc_,* and *id,* *SI Appendix,* Table S2. For each sediment type, we calculated the average values of ∂ using Eq. **2** between adjacent samples in a core in which the sediment type did not change (∂1), and repeated this for instances in which the sediment type did change between adjacent samples (∂2). To find the combination of parameters from Eqs. **5**–**8** that best fit the observed average values of ∂1 and ∂2 we used a Monte Carlo approach in which chains began from random values of *e_1_* and *e_2_* between −1 and 1. For each value of *e1* and *e2* using increments of 0.01 all values of *cl*, *∂xloc* and *id* (ranges 0 to 1, 0 to 2, and 0 to 2 respectively) were explored to calculate values of *∂1* and *∂2* (4 million data points). The closeness of fit of parameters to the observed values was evaluated using Eq. (**9**):

[9] $$fit=\sqrt{(\partial 1_{i}-\partial 1{)}^{2}}+\sqrt{(\partial 2_{i}-\partial 2{)}^{2}}+\sqrt{(\frac{\partial 2_{i}}{\partial 1_{i}}-\frac{\partial 2}{\partial 1}{)}^{2}},$$

where *∂1_i_* and *∂2_i_* are derived from Eqs. **5**–**8** using the *i*th combination of *cl*, *∂_xloc_* and *id* parameters. The parameters associated with the lowest value of fit were stored. The chain progressed *e_1_* and *e_2_* by randomly incrementing or decrementing by 0.01. Consequently, up to 160,000,000,000 data points were explored for each sediment type. The unheated chains were terminated after 20 attempts to improve fit had failed and chains were repeated 1,000 times, so most if not all of the 1.6 × 10^11^ data points were explored. Two independent rounds of Monte Carlo search were attempted to ascertain the stability of the signal, which we found varied little.

### OSL Profiling and Dating (OSL P-D).

High-resolution sediment chronologies were constructed for 22 of the recovered cores using the methodologies described in Kinnaird *et al.* (31). Temporal constraints were provided from detailed luminescence stratigraphies constructed for each investigated core, based on proxy data obtained at sampling (n = 1,104) and calibrated data obtained through subsequent laboratory analyses (n = 949, 86% of sample set). For full technical details the reader is directed to this publication. However, relevant to the discussion on the taphonomy of the sedaDNA, are the luminescence stratigraphies, which contextualize the AMS dates and OSL ages, and provide insight on the depositional histories and mechanisms. These stratigraphies were generated using two approaches: stage 1, during core sampling, relative luminescence stratigraphies were generated from proxy luminescence data generated with portable OSL equipment; and in stage 2, the stratigraphies were “calibrated,” by subjecting subsamples to laboratory luminescence screening and characterization measurements, then constructing apparent dose-depth and sensitivity-depth profiles for each core. Breaks, or step changes, in IRSL and OSL net signal intensities (stage 1) and OSL apparent doses (stage 2) indicate where discontinuities, or unconformities, are present in the core stratigraphies. Further, signal-depth progressions provide insights on the rates of sedimentation: consistent signal intensities or apparent doses with depth indicate high rates of sedimentation, whereas, slow, steady signal-depth progressions represent slow rates of sedimentation. The range in magnitude across these progressions provides the relative rate of sedimentation. It was established that most cores were characterized by coherent stratigraphies, with the exception of ELF2, indicating readily interpretable sequences. Large temporal breaks, related to abrupt erosive events were noted in ELF45, ELF19, ELF39, ELF1A, ELF7, and ELF20.

### Identification of *Pterocarya*.

Members of the Juglandaceae are closely related with major groups diversified within the last few million years (83), and members of *Juglans* and *Pterocarya* are indistinguishable at typical metabarcoding targets such as *trnL* chloroplast. To resolve the identity of the ancient Juglandaceae: Reads that had been assigned to Juglandaceae using MEGAN (78) were used to search against the Juglandaceae section of the Whole Shotgun Genome (WSG) database (NCBI) using BLAST. This returned 206,113 bases from 4,904 reads of sedaDNA aligned to seven species of *Juglans* and one species of *Pterocarya*. We used custom scripts to extract the pairwise alignments from the BLAST output, realign all taxa using MUSCLE (84) and concatenated alignments (with 5 base gap intervals), Dataset S16. At this stage, the alignment contained 8,899 SNP positions, with the largest proportion (271 SNPs) supporting a clade uniting the ancient *Juglandaceae* sequence with *P. stenoptera* (bootstrap support 100%), while the next closest taxon shared 82 SNPs. The alignment was then manually examined for sedaDNA reads that varied from the Juglandaceae genomes and searched against the Nucleotide (NCBI) database and removed if the top matches were not Juglandaceae. This dataset was then used to examine the 5′ and 3′ damage signal for the ancient Juglandaceae using MetaDamage which returned a signal based on 1,133 successfully aligned reads. To remove terminal base modifications typical of ancient DNA 5 bases were clipped from both the 5′ and 3′ ends of the alignments. Reads which aligned to multiple locations in the subject genomes were removed. Singleton sites in the sedaDNA were removed as possible base modifications, leaving 37,210 bases of sedaDNA aligned. The data were then more stringently constrained by removing all phylogenetically informative sites in which sedaDNA showed a transition, resulting in 11 shared SNP positions between *P. stenoptera* and the ancient Juglandaceae sequence out of 300 SNP positions (bootstrap support 77% for clade), fivefold more than SNPs shared with any other taxon. We then removed all sites for the sedaDNA in which data was incomplete across all taxa, leaving 37,163 bases of sedaDNA aligned and 300 SNP positions in which *P. stenoptera* still shared 2 SNP positions with the ancient Juglandaceae, but not with other Juglans species. We used *Carya cathayensis* as an outgroup (85) and generated a maximum likelihood tree using raxml-ng (39), with the General Time Reversible (GTR) model of nucleotide substitution (Fig. 4*B*). At all levels of data filtering and pruning we found overwhelming support for the ancient Juglandaceae signal to form a clade with *P. stenoptera* rather than any other member of the Juglandaceae. We noted that phylogenetically informative sites were from reads recovered from a relatively small number of samples from just two cores where read counts were highest for this taxonomic grouping: almost all came from ELF19-238, ELF19-310, and ELF19-365, while one came from ELF45-252. We considered the possibility that more than one species from this unexpected family would be present at these two sites as unlikely. The remaining sedaDNA reads were not informative within the Juglandaceae, we therefore made the parsimonious assumption that these probably represented the same species across the study site.

## Supplementary Material

Appendix 01 (PDF)

Dataset S01 (XLSX)

Dataset S02 (XLSX)

Dataset S03 (XLSX)

Dataset S04 (XLSX)

Dataset S05 (XLSX)

Dataset S06 (XLSX)

Dataset S07 (XLSX)

Dataset S08 (XLSX)

Dataset S09 (XLSX)

Dataset S10 (XLSX)

Dataset S11 (XLSX)

Dataset S12 (XLSX)

Dataset S13 (XLSX)

Dataset S14 (XLSX)

Dataset S15 (XLSX)

Dataset S16 (TXT)

## Data Availability

DNA sequence data have been deposited in NCBI SRA (PRJNA1001812) (86).
