# Supplementary material for: Early colonization before inundation consistent with northern glacial refugia in Southern Doggerland revealed by sedimentary ancient DNA
Source: Proc Natl Acad Sci U S A. 2026 Mar 10;123(11):e2508402123. doi: 10.1073/pnas.2508402123 (PMC12994208; doi:10.1073/pnas.2508402123)
Supplement: Supplementary file 1 — Appendix 01 (PDF) [file pnas.2508402123.sapp.pdf]

PNAS

Supporting Information for
Pleistocene-Holocene sedaDNA reconstruction of Southern Doggerland reveals early colonization before inundation consistent with northern refugia.

Robin G Allaby[1*], Rosie Ware[1], Rebecca Cribdon[1], Teri A Hansford[1], Tim Kinnaird[2], Derek Hamilton[3], Logan Kistler[4], Phil Murgatroyd[5], Richard Bates[2], Simon Fitch[5], Vincent Gaffney[5]

*Robin G Allaby
Email: r.g.allaby@warwick.ac.uk

This PDF file includes:

Supporting text
Figures S1 to S5
Tables S1 to S3
Legends of Datasets S1 to S16
SI References

Supporting Information Text

A note on the origin of exotic signals in sedaDNA

A small proportion of reads (0.07%) were still assigned to non-European taxa after screening against blanks, as is often observed in sedaDNA studies. These reads show a damage pattern diagnostic for ancient DNA (Supplementary Figure 1), leading us to conclude that these are cases of taxonomic mis-assignation rather than modern contamination from reagents or other sources.

All researchers dealing with sedaDNA are familiar with the retrieval of ubiquitous DNA that appears to be exotic. Some of this signal is attributable to DNA present in reagents used to prepare ancient DNA libraries, the so called 'kitome'[1]. In ancient DNA studies it is standard practice to sequence blank libraries and subtract taxa identified from the taxa identified from ancient DNA samples. However, it is typical for exotic assignations to persist, which on close examination with closest database entries can appear to be genuine instances of the exotic taxa. It is customary practice to exclude such instances of taxa as extraneous sources of contamination, which is a less than satisfactory explanation. Moreover, reagent and other contaminants are from modern sources by definition , and do not show diagnostic ancient DNA degradation as we observe here. Removal of exotic taxa is a reasonable practice, and unexotic assignations have been shown to be assigned with over 95% accuracy with this data set using current pipelines[2], even when the true taxon of origin is absent from the database. However, the origin of the exotic signal has remained persistently enigmatic.

In this study, the exotic signal was subject to a MetaDamage analysis as implemented in Gaffney et al 2020[3] that demonstrated a damage signature consistent with ancient DNA. This suggests that the origin of the signal stems from the ancient DNA itself, and the origin of the signal is likely to stem from the mis-assignation of taxa that are really present. As part of the data reduction analysis for this study, we carried out a pairwise correlation analysis of occurrence between all pairs of taxa, which revealed likely sources for the exotic signal, Supplementary Table 5. The analysis reveals the co-occurrence of exotic taxa with more mundane and expected groups, but also indicates the biases that likely lie behind the generation of the exotic signal. Typically, the exotic taxa are closely associated with more expected species with very large genomes (10-150 Gb), such as those found in various monocotyledon groups, like the Arundinae and Allium groups. In these cases complete genome coverage is unlikely of the true, large genome species in databases leading to sparsely populated database coverage described in Cribdon et al 2020[2]. In such cases it is likely that distantly related species in the database will have the closest matching DNA sequence entry. It is also likely that such assignations will have high read counts by virtue of the fact that organisms with large genomes leave behind more genetic material and are replete with highly repetitive sequence elements. We would therefore caution overly simplistic interpretation of read counts, and the presence of exotics.

Supplementary Table 5 can therefore be used to explore the likely origin of various exotic taxa that will likely be identified in many sedaDNA studies and thus highlight regions which are poorly represented in databases.

A

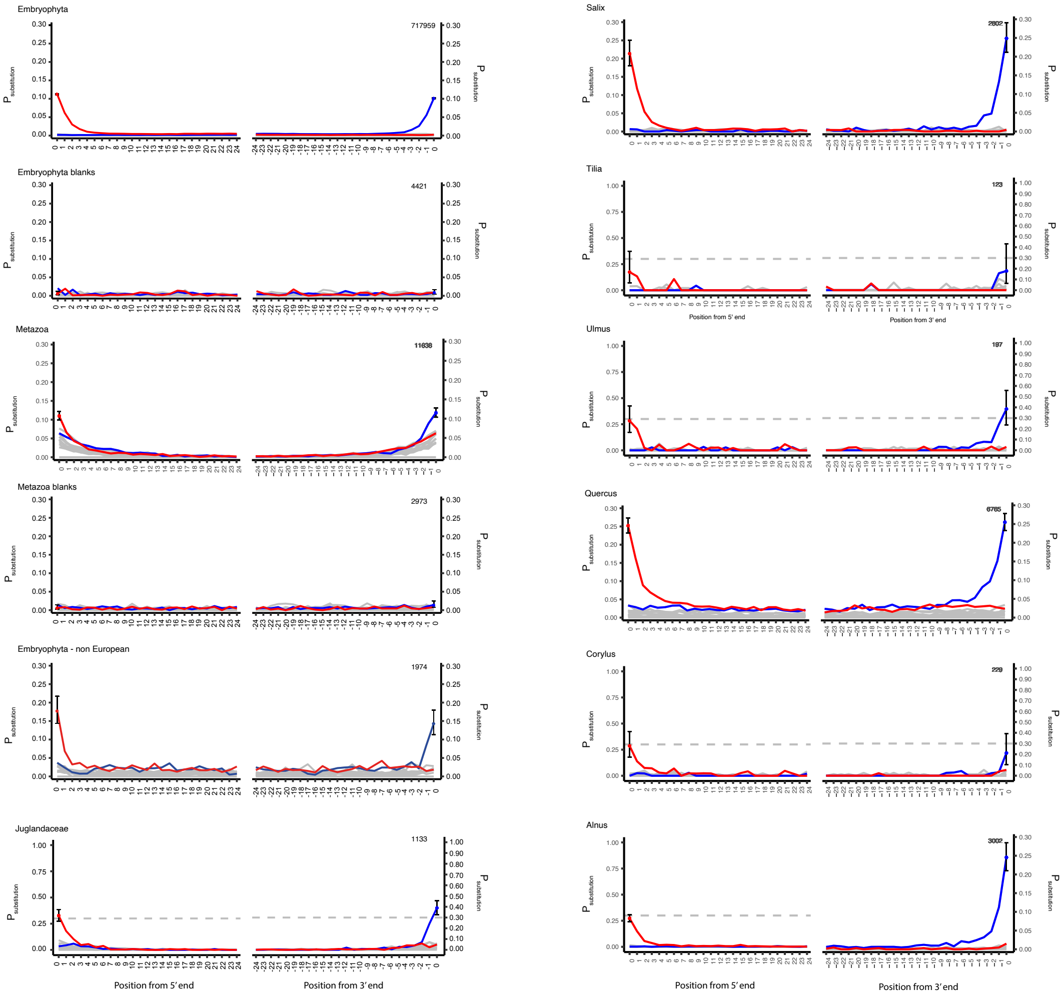

Embryophyta
717959
Embryophyta blanks
4421
Metazoa
11638
Metazoa blanks
2973
Embryophyta - non European
1974
Juglandaceae
1133
Salix
2802
Tilia
123
Ulmus
197
Quercus
6765
Corylus
229
Alnus
3002
P substitution
Position from 5' end
Position from 3' end

B

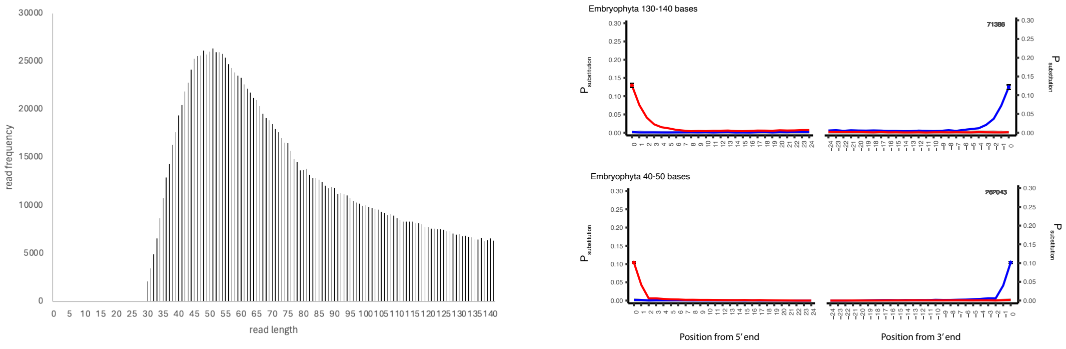

read frequency
read length
Embryophyta 130-140 bases
71386
Embryophyta 40-50 bases
262043
P substitution
Position from 5' end
Position from 3' end

Fig. S1. Base mismatch profiles of sedaDNA. A.Profiles calculated using the MetaDamage approach described in Gaffney *et al.* 2020. X-axis denotes the base position relative to the 5' (left) and 3' (right) end of the molecule. Y-axis shows the probability of a mismatch between a read and the most similar database entry. Red indicates C to T transitions, blue denotes G to A transitions. The number of reads each analysis is based on is given at the top right of each graph. B. Read size distribution of PIA filtered reads. Mismatch profiles are shown for both modal region of distribution (40-50 bases) and top end of the size distribution (130-140 bases).

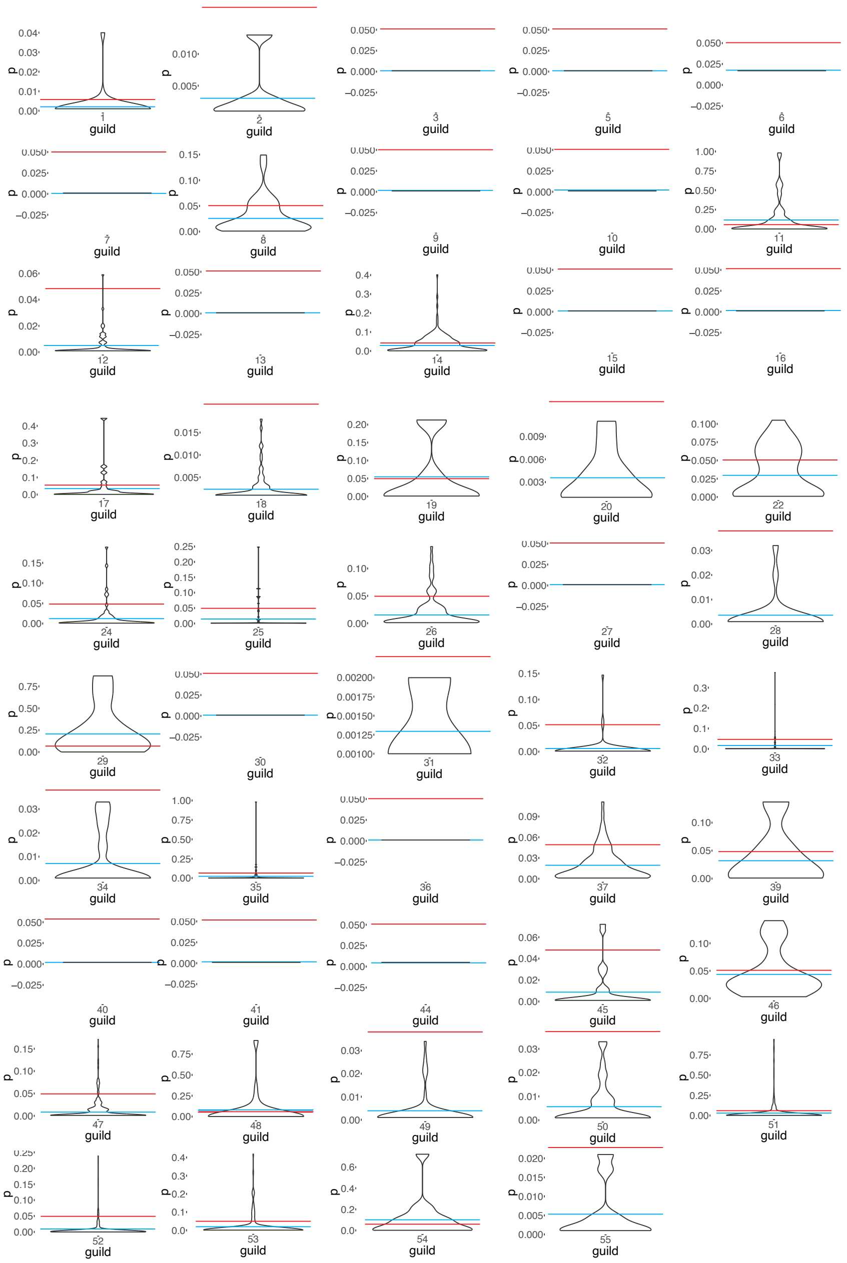

Figure S2. Distributions of pairwise correlation *p* values for each guild. Values of *p* were determined using the manyglm () function of the mvabund R package[83]. The 0.05 threshold is shown by red lines, the mean *p* value of each guild distribution is shown by blue lines.

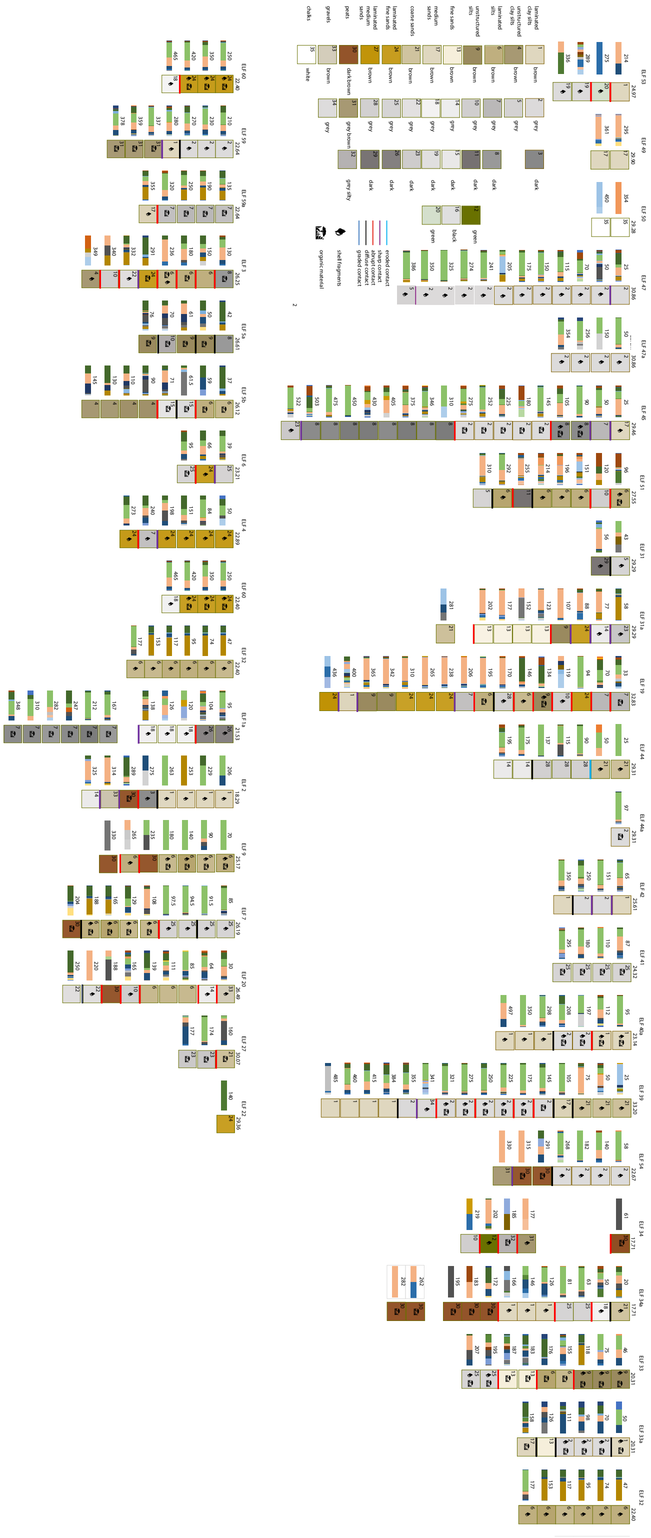

Fig. S3. Core sediment and plant guild profile associations. Depths sample points within cores given above plant guild profile bars in centimetres. Depth of seabed is given in metres above each core profile.

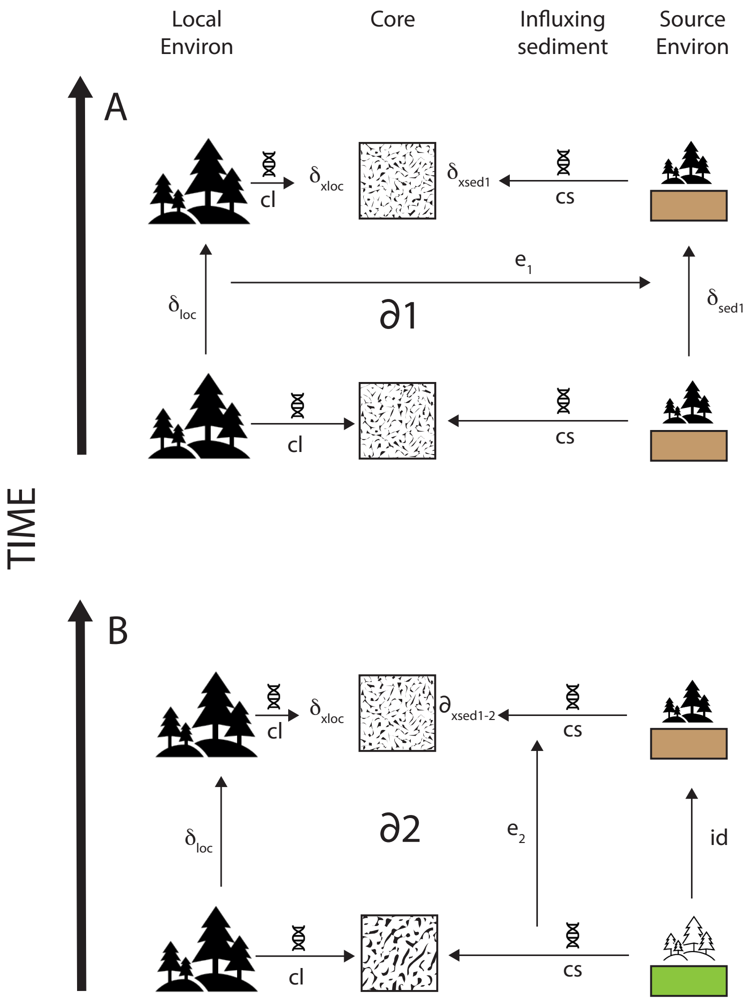

Fig. S4. sedaDNA deposition taphonomy model. A. Adjacent samples of same sediment type 1. B. Adjacent samples of sediment types 1 and 2. Parameters as follows: $\partial 1$ – plant guild distance between adjacent samples of the same type; $\partial 2$ – plant guild distance between adjacent samples of different sediment types; cl – proportion of contribution from local sources; cs – proportion contribution from influxing sediment from source environment; d_{loc} – change in guild profile of local environment over time; d_{xloc} – contribution of d_{loc} to plant guild distance ∂ between samples; d_{sed1} – change in guild profile of influxing sediment source environment over time; d_{xsed1} contribution of d_{sed1} to plant guild distance ∂ between samples; id – plant guild distance between different influxing sediment source environments 1 and 2; $d_{xsed1-2}$ – contribution of id to plant guild distance ∂ between samples; e1 – environmental variable 1 describing the difference in rate of plant profile change over time between dloc and dsed1; e2 – environmental variable 2 describing the difference in influx rates from source environments 1 and 2.

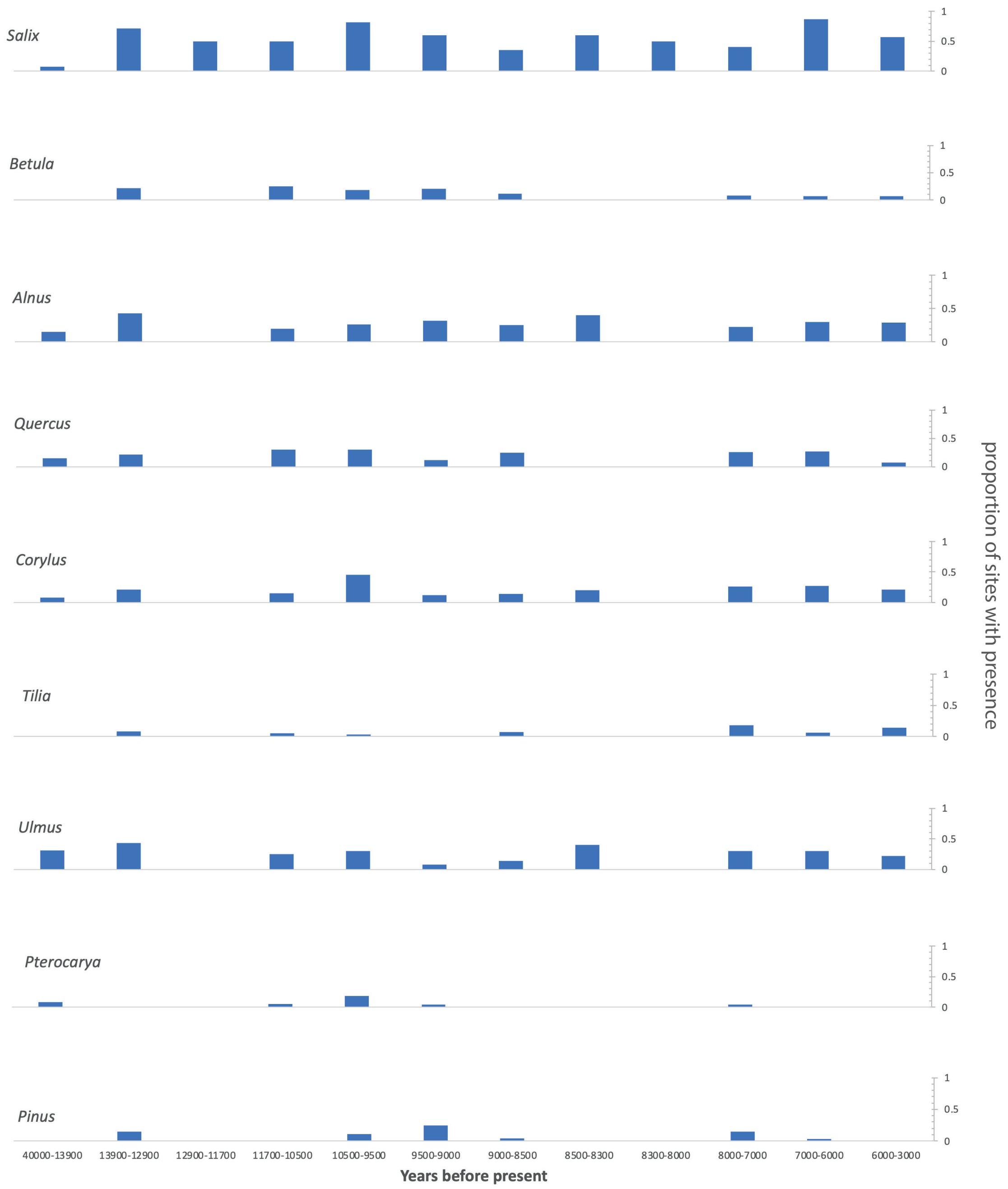

Fig. S5. Frequency of presence of tree species over time. Bars indicate the proportion of sample sites in time periods at which tree species are present.

Table S1 Plant Guild membership

Guild	Number of taxa	Guild influence	mean correlation of taxa	standard deviation of taxa correlations	*mean p value*	Guild nature	Taxa
1	8	1.177677052	0.727333101	0.107087891	0.002642857	wet meadow	Carduinae Caryophyllaceae Plantaginaceae Sanguisorbeae Myrtales Agrostidinae Coleanthinae Polypodiopsida
2	4	0.818942347	0.619822536	0.135239974	0.0035	meadow	Cichorieae Orchidaceae Astereae Solanales
3	2	0.020081432	0.722980669	na	0.001		Gentianeae Jungermanniidae
4	1	0.076665493	na	na	0		Caryophyllales
5	2	0.012864777	0.861504602	na	0.001		Poinae Hypnales
6	2	0.133911384	0.801622766	na	0.017	cold heath	Ericaceae Dryadoideae
7	2	2.873619782	0.866781121	na	0.001	marsh	Phragmites Paniceae
8	9	6.420249549	0.318803295	0.173077945	0.029222222	meadow	Stuckenia Rhamnaceae Mentheae Apiales Anthemideae core genistoids Lythraceae Polypodiidae Polypodiales
9	2	0.004993169	0.580392157	na	0.001	meadow	Veroniceae Helianthemum
10	2	0.109093127	0.434760193	na	0.001	marshy	Cornus Juncaceae
11	15	1.18857322	0.187218164	0.215988224	0.12282857	geranium associated habitats	Typhaceae Typha Salicornioideae Aquifoliales Ilex Berberidaceae Mentha Geranium Neottia Nartheciaceae Aristolochiaceae Artemisiinae Panicum Thelypteridaceae Cephaloziineae
12	13	37.07152466	0.458711776	0.149168988	0.004653846	meadow/marsh	Sparganium Arundinoideae Artemisia Berberidoideae Taraxacum Lobelia Epidendroideae Corallorhiza Caprifoliaceae PACMAD clade Panicoideae Aspleniineae Marchantiophyta
13	7	0.027353011	0.783321163	0.126344524	0.001	Pterocarya pine habitat	Pinaceae Juglans Lathyrus Urticaceae Sanguisorba Deschampsia Equisetaceae
14	21	2.775765829	0.238265438	0.164175395	0.034714286	pond/meadow	Ceratophyllum Ericoideae Caesalpinioideae Sapindaceae Achillea Cirsium Campanula Primula Salvia Solanum Vicia Aroideae Cypripedioideae Calypsoinae Lilium Boraginales Astragalus Avena Elymus Anthocerotophyta Jungermanniales
15	2	0.01264594	0.621315733	na	0.001		Utricularia Marchantiidae
16	6	0.078732394	0.694401141	0.120883947	0.001	meadow	Eleocharis Boraginaceae Gentianaceae Pedicularideae Caltha Pteridoideae
17	8	1.717023814	0.366878778	0.175664908	0.036857143	boggy meadow	Cladium Daucus carota Asphodeloideae Oxalidales Thymelaeaceae Viscaceae Andropogoneae Pteridium
18	13	103.0902641	0.666428527	0.147181453	0.002653846	shallow marine marsh	Alismataceae Sagittaria Lemnoideae Schoeneae Nymphaeales Nymphaeaceae Zosteraceae Zostera Droseraceae Araceae Liliaceae Malvales Liliales
19	5	0.052122744	0.152135075	0.182965735	0.0522	Vaccinium associaed habitats	Cornales Vaccinium Mediterranean clade Amaryllidoideae Jungermanniopsida
20	5	0.624324003	0.438763246	0.121510884	0.0036		Lauraceae Crepidinae Trichosporeae Juncus Dicranidae
21	1	0.00031152	na	na	0		Fabales
22	8	1.306320238	0.26761653	0.143693016	0.033071429	dry meadow	Ziziphus Orobancheae Cactineae Geum Fragaria Fabaceae Papilionoideae Bryidae
23	1	0.002132659	na	na	0		Cistaceae
24	13	1.810650092	0.433178493	0.15617232	0.012217949	Hawthorn habitat	Lentibulariaceae Adoxaceae Viburnum Crataegus Ulmaceae Nolinoideae Iris Neottieae Araliaceae Dipsacales Arecaceae Athyriaceae Phegopteridoideae
25	12	2.203508335	0.56817379	0.289153547	0.015969697	Pine meadow	Daucus Pinidae Pinus Vitaceae Cichorioideae Rubiaceae Cucurbitales Cannabaceae Hordeum Bryophyta Bryophytina Bryopsida
26	14	66.27291314	0.865327045	0.102758059	0.020131868	Willow habitat	Potamogetonaceae Betulaceae Alnus Betula Salicaceae Saliceae Populus Salix Pyroleae Nepetoideae Polygonoideae Persicarieae Asteroideae Malvoideae
27	6	0.446326745	0.815599906	0.095538287	0.001		Crassulaceae Camelineae lamiids Aveninae Marchantiales Apiaceae
28	7	0.048126291	0.80961576	0.134480038	0.003714286	cold heath/scrub	Cupressales Arbutoideae Dryas Galegeae Medicago Linaceae Cheilanthoideae
29	5	3.7941367	0.189381452	0.23862826	0.2436	Pterocarya maple habitat	Cupressaceae Juglandaceae Sapindales Rutaceae Triticeae
30	3	0.171887979	0.460069668	0.027496286	0.001	ash habitat	Oleeae Orchidoideae Gnetidae
31	3	3.429173994	0.855104571	0.025842445	0.001333333		Fagales NPAAA clade malvids
32	12	0.389296514	0.887246155	0.08410957	0.005106061		Characeae Armeria Celastraceae Rubus Pyroloideae Cynoglossoideae Lamioideae Sileneae Potentilleae incertae sedis Sanguisorbinae Santalales Calamagrostis
33	21	12.65772057	0.503604329	0.158411539	0.009795238	temperate tree habitat	Salicornia Chenopodioideae Atripliceae Tamaricaceae Lonicera Corylus Fagaceae Quercus Maleae Tilioideae Tilia Laurales Apieae Iridaceae Piperales Malvaceae Chloridoideae Zoysieae Sporobolus Cenchrinae Pteridaceae
34	6	1.503669226	0.704090688	0.111103792	0.007333333		Taxaceae Rumiceae Brassica Geraniaceae Onagroideae Hordeinae
35	16	19.48001188	0.442344561	0.175978333	0.02015	marsh	Hydrocharitaceae Nymphaea Cymodoceaceae Ruppia Molinieae Chenopodium Salicornia subg. Salicornia Suaeda Plumbaginaceae Limonium Chenopodiaceae Euphorbiaceae Dioscoreales Melanthiaceae Aristolochia Solanaceae
36	2	0.02589899	0.754438895	na	0.001	wet woodland	Oleaceae Iridoideae
37	14	2.156629415	0.849925752	0.120924464	0.019252747	meadow	Oenantheae Primulaceae Rubioideae Orobanchaceae Pedicularis Plantago Potentilleae Fragariinae Arabidopsis Thalictrum Apiineae Gentianales Rosoideae Cyperaceae
38	1	0.002748251	na	na	0		Apocynaceae
39	4	1.920962093	0.252282575	0.091747057	0.037166667	meadow	Cuscuta Amyridoideae Lamiales Marchantiopsida
40	2	0.03452196	0.877884445	na	0.002	meadow	Campanulaceae Hologalegina
41	2	1.112445892	0.757410473	na	0.001	meadow	Trifolieae Rosaceae
42	1	0.196193718	na	na	0		Ericales
43	1	0.006897277	na	na	0	meadow	Anemoneae
44	2	0.009460947	0.241911161	na	0.005	shrubby meadow	Hamamelidaceae Fritillaria
45	7	6.373364417	0.585833647	0.177726334	0.010285714	elm habitat	Amygdaleae Prunus Ulmus Hedera Spiraeeae Amygdaloideae Brachypodium
46	6	19.15374861	0.796822749	0.137338286	0.045866667	grassland	Menyanthaceae Apioideae Scutellaria Poaceae BOP clade Polytrichaceae
47	23	7.052558971	0.83742533	0.273246987	0.012721344	pond/meadow	Potamogeton Puccinellia Galium Potentilla Saxifragaceae Papaver Ranunculoideae Delphinieae Ranunculeae Ranunculus Thalictroideae Asteraceae Gnaphalieae Lamiaceae Stachydeae Polygonaceae Loteae Rosoideae incertae sedis Ranunculales Ranunculaceae Poeae Chloroplast Group 2 (Poeae type) Equisetum Pottiaceae
48	11	4.217240731	0.08046662	0.228864926	0.059127273	shrubby heath associated habitat	Oryzeae Suaedoideae Empetrum Vaccinioideae Vaccinieae Anacardiaceae Lotus Actaea Acrogymnospermae Triticodae Dryopteris
49	13	1.145995529	0.229657722	0.317273111	0.003128788	alpine meadow associated habitat	Lycopus Ephedra Empetreae IRL clade Arabideae Brassiceae Brassica oleracea Allium 50 kb inversion clade Brassicaceae Proteales Porellales Lejeuneaceae
50	9	1.462532656	0.889527165	0.066372882	0.006194444	wet meadow	Haloragaceae Myriophyllum Chara Cochlearieae Tussilagininae Veronica Fabeae Senecioneae Loliinae
51	32	4.74950041	0.022856825	0.156384114	0.026752016	vetch and fern associated habitats	Marsileaceae Nuphar Fuireneae Bolboschoenus Cochlearia Lycium Castanea Pyrus Vitis Chrysanthemum Amaranthus Silene Cucurbitaceae Lepidium Agavoideae Convolvulaceae Phaseoleae Agrimoniinae Cypereae Cyperus Stipa Secale Setaria Ophioglossidae Cystopteris Thelypteris Thelypteris palustris Pteridium aquilinum Polypodiineae Dryopteridaceae Dryopteridoideae Polypodiaceae
52	30	2.586869235	0.871464496	0.248558823	0.005391129	wet meadow & trees	Eriophorum Pinus apioid superclade Carduoideae Cardueae Centaureinae Centaurea Rubieae Rhinantheae Euphrasia Rhinanthus Alsineae Persicaria Trifolium Draba Saxifraga Papaveroideae Caltheae Caltha palustris Polygoneae Cyperoideae Carex Scirpeae Oryzoideae Pooideae Poeae Poeae Chloroplast Group 1 (Aveneae type) Festuca Poa Hypnanae
53	20	2.895119625	0.160627192	0.285355019	0.017104762	wet meadow & tree associaed habitat	Allioideae Solanoideae C4 Cyperus incertae sedis Isoetes Posidonia Rhamneae Malus Rosa Dysphania Tofieldiaceae Amaryllidaceae Hyacinthaceae Crocoideae Dioscoreaceae Zygophyllales Stipeae Aristideae Asplenium Polypodioideae Bryaceae
54	6	0.720524169	0.145458639	0.254710181	0.108	fern woodland associated habitat	Najas Quercus robur Malaxidinae Athyrium Blechnaceae Dennstaedtiaceae
55	5	0.372362787	0.520276873	0.1153982	0.0054		Asparagaceae Dioscorea Colchicaceae Brassicales Thelypteridoideae
56	1	0.001810612	na	na	0	meadow	Filipendula

Table S2 Local and influxing sediment contributions to sedaDNA signal

	cl	*cs*	$∂_{xloc}$	$∂_{xsed}$	$∂_{sed1\text{-}2}$	*id*	*(1+e1)*	*(1+e2)*	∂1	∂2	fit	N1	N2	ratio (∂2/∂1)
**sediment combinations**														
all sediments	0.96	0.04	0.2	0.0098408	0.09134587	1.87	1.2723	1.558	0.20987276	0.29139128	8.23E-05	75	44	1.3884188
silts	0.97	0.03	0.18	0.01086079	0.05987035	1.73	1.33	0.2669	0.19085395	0.23986324	2.17E-05	47	24	1.256789487
sands	0.95	0.05	0.24	0.01161607	0.10178576	1.93	0.7335	1.0954	0.25161588	0.34178475	4.20E-06	26	19	1.358359202
clay silts	0.95	0.05	0.16	0.01351327	0.02125274	1.97	1.5252	0.8566	0.1735089	0.18124622	2.22E-05	25	11	1.044593228
non clay silts	0.89	0.11	0.18	0.03055788	0.10945246	1.99	0.3154	1.0534	0.21056424	0.28946226	2.12E-05	22	13	1.374698077
laminated silts	0.98	0.02	0.19	0.00565666	0.07019096	1.93	1.3364	1.2331	0.19566623	0.26020191	2.95E-05	40	17	1.329825361
unstructured silts	0.97	0.03	0.08	0.00258727	0.05818742	1.99	0.2665	1.6707	0.08257781	0.1381699	4.74E-05	2	4	1.673208598
fine sand	0.98	0.02	0.15	0.00572754	0.05566677	1.91	1.7218	1.5165	0.15572061	0.20565963	2.70E-05	17	11	1.320696269
medium sand	0.85	0.15	0.15	0.04270292	0.44963658	1.8	1.1856	1.5934	0.19268852	0.59959364	6.71E-05	4	5	3.111724815
coarse sand	0.3	0.7	0.24	0.9433107	0.25665124	1.93	0.3114	1.6407	1.18329519	0.49666208	4.10E-05	4	3	0.419727965
laminated sands	0.83	0.17	0.1	0.02751059	0.23034619	1.97	0.7182	1.1283	0.12750505	0.33033684	5.42E-05	16	7	2.590774565
unstructured sands	0.34	0.66	0.27	0.20320449	0.0998261	1.87	0.2267	0.2929	0.47319382	0.36982104	2.27E-05	10	12	0.781542406
fine sands (unstructured)	0.37	0.63	0.13	0.19222987	0.09312754	1.92	1.2447	1.8688	0.32225045	0.22313632	4.63E-05	4	5	0.69243137
medium and coarse sands (unstructured)	0.33	0.67	0.12	0.45382762	0.35460217	0.46	1.4018	1.6365	0.57382274	0.47459584	1.52E-05	6	7	0.827077434
**Individual sediment types**														
brown clay laminated silt	0.99	0.01	0.12	0.00161365	0.0302002	1.99	0.6269	1.7049	0.12160778	0.1501916	2.56E-05	4	5	1.235049261
grey clay laminated silt	0.91	0.09	0.18	0.0053876	0.01397501	1.8	1.5815	0.7276	0.18538728	0.19397742	1.75E-05	20	5	1.046336183
brown clay silt unstructured	0.93	0.07	0.13	0.01355863	0.14288374	1.73	1.6593	1.5205	0.14354579	0.27286334	6.12E-05	1	1	1.900880198
brown laminated silt	0.95	0.05	0.22	0.01448779	0.17927837	1.89	1.2206	1.1435	0.23447852	0.3992648	3.24E-05	11	6	1.702777745
grey laminated silt	0.79	0.21	0.06	0.00572305	0.25202999	1.99	1.2569	0.1395	0.06570498	0.3119362	0.00023334	5	3	4.747527756
dark grey laminated silt	0.29	0.71	0.07	0.27062362	0.08180872	0.1	1.9517	1.4969	0.34060299	0.15179574	4.47E-05	5	1	0.445667662
brown grey silt unstructured	0.94	0.06	0.02	0.00161088	0.073276	1.81	1.1464	1.131	0.02160983	0.09327209	3.39E-05	1	3	4.316187419
brown grey sand fine	0.41	0.59	0.11	0.29255012	0.01333223	1.98	0.9538	1.7905	0.40257146	0.12333333	3.59E-05	3	2	0.306363811
grey sand fine	0.9	0.1	0.07	0.01128457	0.21965611	1.75	1.3976	1.7959	0.08128741	0.28967165	8.50E-05	1	3	3.563548885
grey sand medium	0.45	0.55	0.11	0.13171912	-0.0283687	1.93	1.1444	1.8513	0.24172163	0.08162621	3.20E-05	1	2	0.337686829
brown/green sand medium	*nd*	*nd*	*nd*	*nd*	*nd*	*nd*	*nd*	*nd*	0.00292078	0.83446609	*nd*	1	2	285.6996943
grey sand coarse	0.02	0.98	0.03	1.39503889	0.03514685	1.34	1.736	1.8537	1.42526008	0.06505102	0.00039134	1	1	0.045641509
dark grey sand coarse	0.49	0.51	0.32	0.62133062	0.39246109	1.91	1.2496	1.2354	0.9413303	0.71246761	1.40E-05	1	2	0.756873132
brown grey sand fine laminated	0.97	0.03	0.1	0.00538723	0.09425914	1.99	0.5951	0.1301	0.1053892	0.19426974	7.87E-05	6	4	1.843355331
grey sand fine laminated	0.8	0.2	0.09	0.00598306	0.09475806	1.99	0.5636	1.5051	0.09597609	0.18474767	4.90E-05	7	2	1.924934354
grey sand medium laminated	0.67	0.33	0.17	0.09305069	0.99577678	1.94	1.7119	1.3442	0.26305583	1.16578361	7.27E-05	2	1	4.431696548
grey brown peat	0.52	0.48	0.04	0.06157084	0.04549222	0.08	1.7722	1.3466	0.10156008	0.08548468	3.32E-05	2	1	0.841715322

Table S3. Contextual anchors of undated sediments to dated sediments by plant guild similarity and proximity

Contextual sample	Anchor sample	phylogenetic distance	date (yrs BP)
ELF47A 354	ELF51 292	0.02488644	>13310
ELF47A 256	ELF45 522	0.03265432	>11010
ELF47A 150	ELF47A 256	0.18518519	>11010
ELF47A 50	ELF45 90	0.00567716	>7440
ELF44 195	ELF39 460	0.0140065	>13040
ELF44 175	ELF39 225	0.0020939	<7710
ELF44 137	ELF54 58	0.00601448	6000–5230 (95% range age modelled date)
ELF44 115	ELF34A 63	0.06933464	circa 6000
ELF44 90	ELF39 105	0.00245665	<7710
ELF44 50	-	-	-
ELF44 25	-	-	-
ELF44A 97	ELF39 175	0.00188801	<7710
ELF41 295	ELF39 145	0.03807077	<7710
ELF41 180	ELF39 145	0.03998565	<7710
ELF41 110	ELF39 105	0.01549899	<7710
ELF41 87	ELF39 50	0.04581544	<7710
ELF40A 487	-	-	-
ELF40A 350	ELF19 94	0.00132605	>5970
ELF40A 298	ELF42 350	0.00472132	<5750
ELF40A 208	-	-	-
ELF40A 197	-	-	-
ELF40A 112	ELF42 65	0.02721758	<4510
ELF40A 95	-	-	-
ELF60 465	ELF54 140	0.00465348	6145–5365 (95% range age modelled date)
ELF60 420	ELF59 337	0.00150836	<7120, >6900
ELF60 350	ELF59 280	0.01449371	<6900
ELF60 250	ELF59 230	0.00547658	<6900
ELF59A 355	ELF3 332	0.03134108	>10710
ELF59A 320	-	-	-
ELF59A 250	ELF3 332	0.1344078	>10710
ELF59A 190	ELF3 332	0.03699522	>10710
ELF59A 135	ELF3 332	0.04781934	>10710
ELF6 95	ELF59 359	0.03236739	>7120
ELF6 66	ELF3 180	0.03129626	<7950
ELF6 39	ELF1A 95	0.03790851	<6030
ELF4 273	EF1A 348	0.03744898	7189
ELF4 240	-	-	-
ELF4 198	ELF1A 104	0.16502812	<7160
ELF4 151	ELF1A 104	0.06541377	<7160
ELF4 84	ELF1A 95	0.02450715	<6030
ELF4 50	-	-	-

Dataset S1 (separate file). Dataset S1. Filtered plant read counts in cores 1-60 general scan.

Dataset S2 (separate file). Dataset S2. Filtered plant read counts in depth on selected cores.

Dataset S3 (separate file). Dataset S3. Filtered animal read counts in cores 1-60 general scan.

Dataset S4 (separate file). Dataset S4. Filtered animal read counts in depth on selected cores.

Dataset S5 (separate file) Dataset S5 MetaDamage estimates of base modification

Dataset S6 (separate file) Dataset S6 Mean read length and read size distributions for assigned taxa

Dataset S7 (separate file). Dataset S7. Pairwise correlation coefficients of co-occurrence between taxa.

Dataset S8 (separate file). Dataset S8. Proportion Guild membership of sedaDNA samples.

Dataset S9 (separate file). Dataset S9. Pairwise distances between guild structures of samples by difference in sum of squares (values 0-2).

Dataset S10 (separate file). Dataset S10. Probabilities of taxa from adjacent core samples being drawn from the same underlying distribution. Lowest probabilities shown. Grey indicates insufficient data for this analysis.

Dataset S11 (separate file). Dataset S11. Percentage contribution of read counts explicable by diffusion between adjacent samples.

Dataset S12 (separate file). Dataset S12. OSL and AMS C14 dates of samples.

Dataset S13 (separate file). Dataset S13. Guild presence in secure sediments unlikely to be affected by sediment influxes.

Dataset S14 (separate file). Dataset S14. Species presence in secure sediments unlikely to be affected by sediment influxes.

Dataset S15 (separate file). Dataset S15. Cores and sample dates in which the presence of Pterocarya pollen has been recorded in the Southern Doggerland area.

Dataset S16 (separate file). Dataset S16. SedaDNA reads affiliated with Juglandaceae aligned to Juglandaceae genomic DNA.

SI References

1.Salter SJ, Cox MJ, Turek EM, Calus ST, Cookson WO, Moffatt MF, Turner P, Parkhill J, Loman NJ, Walker AW. Reagent and laboratory contamination can critically impact sequence-based microbiome analyses. BMC Biol. 2014;12:87.

2. Cribdon, B., Ware, R., Smith, O., Gaffney, V., Allaby, R.G. PIA: More accurate taxonomic assignment of metagenomic data demonstrated in sedaDNA from the North Sea. *Front. Ecol. Evol.* **8**, 84 (2020).

3. Gaffney V, Fitch S, Bates M, Ware RL, Kinnaird T, Gearey B, Hill T, Telford R, Batt C, Stern B, Whittaker J, Davies S, Ben Sharada M, Everett R, Cribdon R, Kistler L, Harris S, Kearney K, Walker J, Muru M, Hamilton D, Law M, Finlay A, Bates R, Allaby RG. Multi-proxy characterisation of the Storegga tsunami and its impact on the early Holocene landscape of the southern North Sea. *Geosciences* **10**, 270 (2020)
